# Resonance peak extraction method based on human ear model and its application in bearing fault diagnosis

**DOI:** 10.1038/s41598-025-25794-8

**Published:** 2025-11-25

**Authors:** Yongming Zhao, Yungong Li, Chao Wang, Shiyu Yan, Jiazhuang Dong, Jinxin Yang

**Affiliations:** School of Mechanical Engineering and Automation, Northeastern University, Shenyang, 110089 Liaoning China

**Keywords:** Fault diagnosis, Auditory, Resonant peak, Signal processing, Random forest, Engineering, Mathematics and computing

## Abstract

Rolling bearings are crucial in rotating machinery, and combining natural and characteristic frequencies improves fault detection. However, natural frequencies face challenges like feature extraction difficulties and drift, necessitating resonance peak information supplementation. Existing methods for extracting resonance peaks often struggle with low quality, false peaks, and merging issues. This paper introduces a novel resonance peak extraction method based on auditory saliency (RESAS), inspired by the human auditory system. RESAS combines Gammatone filtering, multi-scale Gaussian filtering, and lateral inhibition to simulate auditory attention and efficiently extract resonance peaks. A resonance peak saliency map (RPSP) is generated, from which features are extracted and used as input to an improved random forest model (TF-RF) for fault classification. Tests on the QPZZ-II Fault Simulation Test Bench and KWCU data show that the method effectively identifies bearing faults at various speeds and loads, demonstrating its strong potential for application. Furthermore, due to its broadband characteristics and capacity to excite the system’s natural frequencies, this method has potential for scalability to other impact-type fault systems.

## Introduction

When a bearing rotates at a constant speed, the presence of defects induces periodic impulse response vibrations^1,2^. Faults occurring in different components of the bearing are manifested at their respective impact response frequencies–referred to as characteristic frequencies–which appear at distinct rates^3,4^. For example, VGS A et al. constructed a failure model for deep groove ball bearings in their article and conducted a response analysis to verify this point^5^. Currently, extracting impulse response components from signals and calculating the corresponding characteristic frequencies has remained a central objective in the diagnosis of rolling bearing faults. Methods such as the Hilbert-Huang Transform (HHT)^6,7^, Empirical Mode Decomposition (EMD)^8^, and Continuous Wavelet Transform (CWT)^9,10^ have demonstrated significant practical effectiveness in achieving this goal. However, these methods suffer from significant limitations in real-world applications. First, when the defect is small, the impulse response components are difficult to extract effectively, thereby reducing diagnostic accuracy^11^. Second, as the rolling elements shift position during operation, deviations arise between the actual and theoretical characteristic frequencies^12^. Third, under strong noise conditions, weak fault features are often masked by noise, making accurate extraction challenging^13^. Therefore, to enhance the accuracy of rolling bearing fault classification, it is essential to incorporate various signal features that can complement and support characteristic frequencies. In recent years, researchers have explored intelligent diagnostic technologies based on modulated signal analysis and machine learning^14–16^, as well as methods such as deep independent component analysis and variational modal decomposition, which are effective in extracting perceptually significant features from noisy environments^17^. Additionally, psychoacoustic features, particularly those related to sound quality metrics, have shown promising effectiveness in multi-fault diagnosis of motors and bearings^18^. These features leverage perceptual properties of sound, such as frequency, intensity, and temporal patterns, to provide complementary insights for fault detection. However, psychoacoustic methods can be limited when dealing with complex vibration signals and background noise, which has led to the development of alternative or supplementary feature extraction strategies.

When a fault occurs in a rolling bearing, the system’s natural frequency changes accordingly^19,20^. The resulting impact signals exhibit broadband energy that excites resonance peaks, whose shapes and shifts provide reliable fault indicators^21^. Even weak impulses can generate observable resonance peaks, which remain largely insensitive to rotational speed and have been successfully used for fault classification^22^. To extract such features, traditional methods such as Short-Time Fourier Transform (STFT)^23^, Cepstrum Analysis^24^, and Linear Predictive Coding (LPC)^25^ have been employed. However, their effectiveness is often constrained by spurious peaks caused by noise, the merging of adjacent peaks, and frequency shifts in high-order modes^26,27^. In recent years, artificial intelligence methods, including Elman neural networks and long short-term memory (LSTM) models, have shown promising improvements in the robustness of resonance-based fault classification^28^.

The auditory saliency of the human ear offers a novel perspective for addressing this problem. Auditory saliency refers to the phenomenon in which certain components of a sound signal stand out perceptually from the background due to their physical properties–such as frequency, intensity, or rhythm–or their interaction with the auditory system, thereby capturing the listener’s attention^29^. Owing to their concentrated energy, resonance peaks can exceed the masking threshold of background noise and be sharply perceived by the human ear. In everyday contexts, the human auditory system demonstrates short-term, efficient, and accurate perception of resonance peaks, particularly those present in speech signals. Therefore, by modeling the auditory processing mechanism of the human ear and leveraging auditory saliency, it becomes feasible to achieve effective extraction of resonance peaks.

From a physiological standpoint, when external sounds enter the auditory system, they sequentially traverse the outer ear, middle ear, and inner ear before being transmitted to the brain via the auditory pathway. The outer ear primarily functions to collect and direct sound waves, while the middle ear transforms them into mechanical vibrations and amplifies their intensity. Critical stages of signal processing and suppression occur in the inner ear and auditory pathway. Within the cochlea, hair cells exhibit frequency-specific selectivity by responding to particular sound frequencies according to the frequency-dependent vibrations of the basilar membrane. Moreover, the basilar membrane attenuates adjacent-frequency interference and high-frequency noise through mechanical damping, thereby enabling smoother signal representation. In the auditory pathway, neurons further refine spectral resolution by employing lateral inhibition, which suppresses responses from neighboring frequencies, sharpens spectral boundaries, and enhances the contrast between resonance peaks and valleys^30,31^. Taken together, these biological mechanisms provide a theoretical and physiological foundation for resonance peak detection. By modeling the auditory processing functions of the inner ear and auditory pathway, and by exploiting the principle of auditory saliency, a biologically inspired and theoretically grounded approach can be developed for the effective extraction of resonance peaks in bearing fault diagnosis.

Inspired by auditory processing mechanisms, this study proposes a resonance peak extraction method grounded in auditory saliency, termed RESAS. The method is designed to emulate key functional stages of the human auditory system for effective resonance peak detection. Specifically, a Gammatone filter bank is first employed to simulate the frequency-specific selectivity of cochlear hair cells. Subsequently, multi-scale Gaussian filtering is applied to mimic the smoothing and noise-suppression functions of the basilar membrane. Finally, a lateral inhibition matrix is constructed to replicate the inhibitory interactions among neurons in the auditory pathway, thereby enhancing the contrast between resonance peaks and valleys. This process yields a resonance peak saliency map (RPSP), which serves as the primary feature representation for rolling bearing fault detection. To enable accurate classification and diagnosis, a Random Forest (RF) model is introduced^32^. RF, as an ensemble learning algorithm, builds multiple decision trees and aggregates their outputs to perform classification and regression tasks with improved stability and robustness. To further enhance computational efficiency and feature discrimination, an improved decision tree framework, denoted as TF-RF, is proposed. Features extracted from the RPSP are utilized as inputs to the TF-RF model for rolling bearing fault classification and diagnosis. Comparable approaches have also been explored in prior studies, where machine learning models were applied to predict vibration characteristics of bearings and to analyze dynamic variations in bearing parameters under complex operating conditions, such as those found in steam turbine systems^33^.

The remainder of this paper is structured as follows. Section 2 presents the proposed RESAS method in detail. Section 3 describes the TL-RF classification framework. Section 4 reports the experimental validation using laboratory measurements based on the KWCU dataset. Section 5 encapsulates the essential results and offers the concluding perspectives of the work.

## Resonance peak extraction system based on auditory saliency (RESAS)

**Fig. 1:**
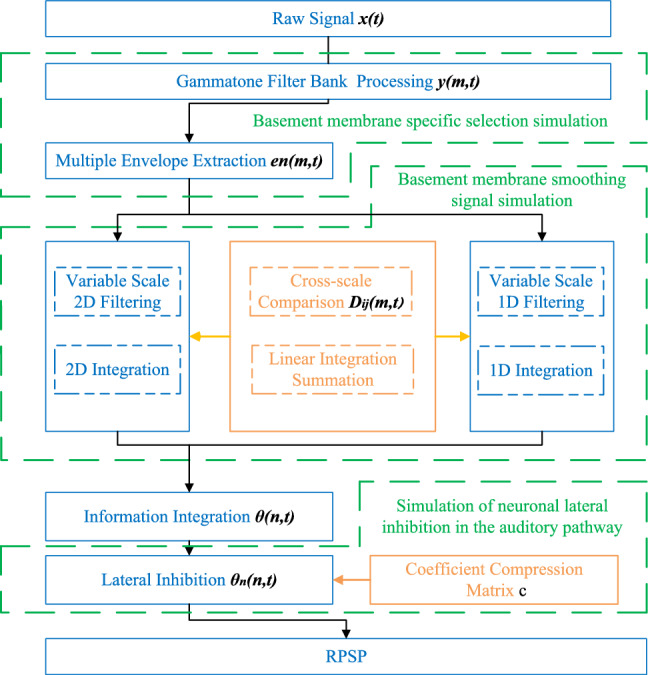
RESAS flow chart.

Inspired by the auditory attention mechanism of the human ear, we developed a novel model called RES AS by simulating different structures of the human ear to achieve similar functionalities. The model first employs Gammatone (GT) filtering and envelope extraction to simulate the frequency-selective characteristics of the basilar membrane. Subsequently, multi-scale two-dimensional and one-dimensional filtering is applied to emulate the membrane’s smoothing effect on auditory signals. Finally, a lateral inhibition matrix is introduced to mimic the lateral inhibition process observed in auditory pathway neurons. The overall procedure is illustrated in Fig. 1.

### Basement membrane specific selection simulation

**Fig. 2:**
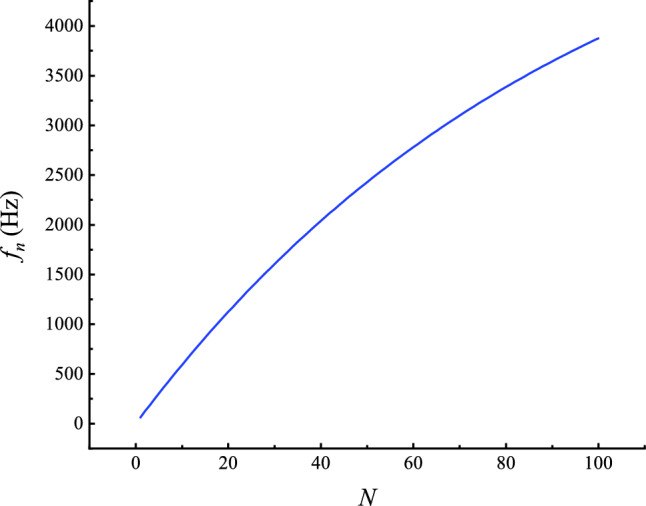
Center frequency distribution.

The Gammatone filter bank exhibits a frequency response that closely aligns with the selective characteristics of basilar membrane hair cells in the human auditory system, thereby enabling an effective approximation of the ear’s frequency resolution and perceptual properties with strong robustness to noise^34^. The filter bank possesses the inherent capability to perform localized analysis in both temporal and spectral domains, which facilitates the attenuation of high-frequency random noise components beyond the target frequency bands. Simultaneously, its energy-concentration property enhances the relative dominance of principal signal components over low-amplitude interference, thus improving the signal-to-noise ratio and effectively suppressing extraneous noise. Assuming the total number of filters is N, the time-domain function of the nth filter is defined as:

1 $$\begin{aligned} h ( n , t ) = B ^ { a } t ^ { a - 1 } e ^ { - 2 \pi B t } \cos ( 2 \pi f _ { n } t + \phi _ { n } ) \end{aligned}$$

where $f _ { n }$ is the center frequency of the nth filter; *B* is the bandwidth parameter; *a* is the filter order, and in this paper 4 is used^35^, $\phi _ { n }$ is 0; The *B* represents the bandwidth selected by the filter, which plays a critical role in determining its attenuation rate and the length of its impulse response. The larger the* B* value, the wider the bandwidth, the faster the decay, and the shorter the duration of the impulse response. It is defined as:

2 $$\begin{aligned} B = 1 . 1 0 9 E R B ( f _ { n } ) = 1 . 1 0 9 \left( 2 4 . 7 + \frac{ 0 . 1 0 8 }{ n } \right) \end{aligned}$$

where ERB ($E R B ( f _ { n } )$) is the equivalent rectangular bandwidth of the nth filter. The center frequencies of the *N* filters are logarithmically distributed along the frequency axis. As the frequency increases, the distribution becomes progressively sparser. The center frequency of the n-th filter is defined as follows, as illustrated in Fig. 2:

3 $$\begin{aligned} f _ { n } = - 1 0 + ( 0 . 5 f _ { h } + 1 0 ) e ^ { - 0 . 1 0 8 N *v } \end{aligned}$$

where $f _ { h }$ is the center frequency of the filter, which is generally taken as 0.5 times the sampling frequency;$f _ { n }$ is the sampling frequency; *v* is the overlap factor of the filter.

Furthermore, a certain degree of overlap $v$ exists between filters, and proper overlap must be ensured to maintain the quality of sampling. The overlap factor $v$ is defined as:

4 $$\begin{aligned} v = \frac{ 2 ( \ln ( f _ { h } - 2 0 - \ln 1 0 ) ) }{ M } \end{aligned}$$

Next, the input signal is filtered. Assuming that the set of *N* filters processes the input signal *x*(*t*) ,the response of the n-th filter is defined as:

5 $$\begin{aligned} y ( n , t ) = x ( t ) \otimes h ( n , t ) \end{aligned}$$

where *x*(*t*) is the signal; $\otimes$ is the convolution symbol indicating that a convolution operation is performed; *y*(*n*, *t*) represents the time domain response of the n-th filter. During the filtering process, phase differences may occur between the outputs of different filters. To eliminate these differences and prevent them from affecting subsequent signal processing and analysis, the following formula can be used^36^:

6 $$\begin{aligned} y ( n , t ) = F ^ { - 1 } \left[ \frac{ X ( f ) \left[ H ( n , f ) \right] }{ \max H ( n , f ) } \right] \end{aligned}$$

where $F ^ { - 1 }$ is the Fourier inverse transform; *X*(*f*) is the frequency-domain expression for *x*(*t*); *H*(*n*, *f*) is the frequency-domain expression for *h*(*n*, *t*).

Cochlear hair cells and sensory neurons respond only to the positive peaks of the signal^37^, allowing them to effectively utilize the envelope information for advanced processing. Therefore, this paper adopts a similar envelope method. First, the filtered signal is discretized, removing the mean value of the entire signal. Then, the orthogonal zero-crossings of each filtered channel signal (the points where the signal crosses zero upwards) are determined, and the peaks between adjacent zero-crossings are identified. A cubic spline interpolation method is used to envelope the peaks. If the function *S*(*x*) satisfies:

1. On each subinterval, *S*(*x*) is a cubic polynomial.
2. *S*(*x*) is twice continuously differentiable over the interpolation interval.
3. *S*(*x*) satisfies the interpolation condition $S ( t _ { k } ) = x ( t _ { k } ) = t _ { k } ( k = 1, 2, 3, \cdots , K )$.

**Fig. 3:**
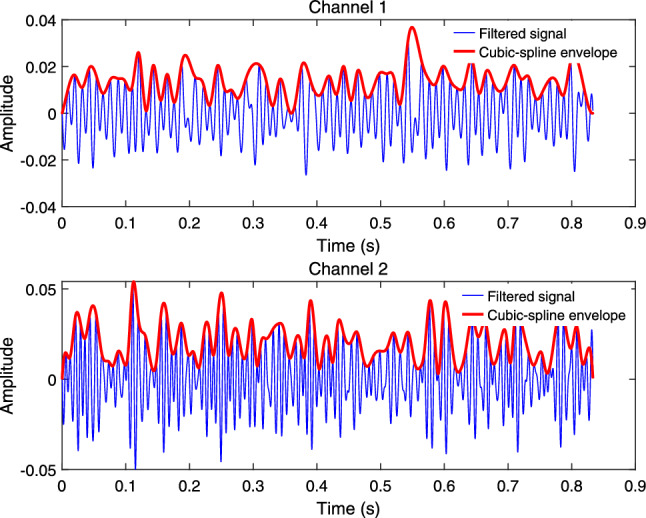
Envelope effect.

*S*(*x*) is the cubic spline interpolation function of $x ( t _ { k } )$. By performing cubic spline interpolation on the peak values, the envelope signal *en*(*n*, *t*) of the signal is obtained. The first filter channel and the second filter channel are shown in Fig. 3. Additional iterations can be performed if higher signal quality is required.

### Basement membrane smoothing signal simulation

**Fig. 4:**
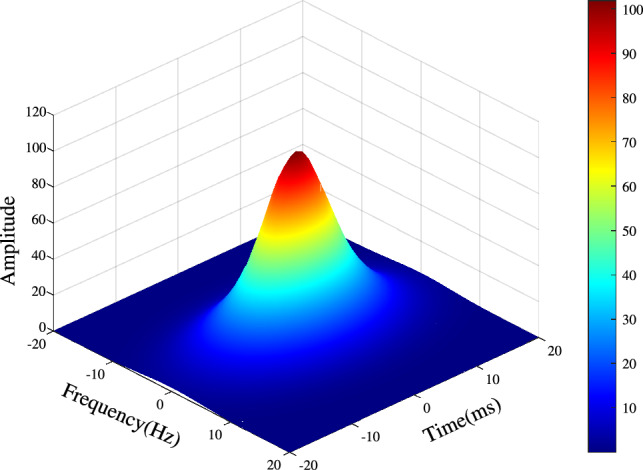
Two-dimensional Gaussian filter shape.

The basilar membrane of the human ear attenuates neighboring frequency crosstalk and high-frequency noise through mechanical damping during frequency decomposition, resulting in a smoother signal and a more prominent main-frequency component^38^. To simulate this mechanism, scale-variant 2D Gaussian filters and scale-variant 1D Gaussian filters are employed for filtering, respectively. Subsequently, the filtering results are integrated to simulate their functions. The two-dimensional Gaussian filter function used in this paper is defined as follows:

7 $$\begin{aligned} g_{(\sigma _{f_1}, \sigma _{t_1}, \sigma _{f_2}, \sigma _{t_2})}(v_f, v_t) = \frac{1}{2\pi (\sigma _{f_1} \sigma _{t_1} \sigma _{f_2} \sigma _{t_2})} \Bigg [ \exp \Big (-\frac{v_f^2}{2\sigma _{f_1}^2} - \frac{v_t^2}{2\sigma _{t_1}^2}\Big ) + \exp \Big (-\frac{v_f^2}{2\sigma _{f_2}^2} - \frac{v_t^2}{2\sigma _{t_2}^2}\Big ) \Bigg ] \end{aligned}$$

where: $v_f$ and $v_t$ denote the frequency and time offsets from the filter center, respectively; $\sigma _{f_1}, \sigma _{f_2}$ represent the frequency scales; $\sigma _{t_1}, \sigma _{t_2}$ represent the time scales. The scale parameters in frequency and time domains are defined as:

8 $$\begin{aligned} \sigma _{f_i} = \frac{m_i}{\sqrt{-\ln (0.01)}}, \qquad \sigma _{t_i} = \frac{n_i}{\sqrt{-\ln (0.01)}}, \quad i = 1, 2,... \end{aligned}$$

where $m_i$ and $n_i$ are the normalized scale factors controlling the bandwidth in frequency and time, respectively. The constant $0.01$ determines the attenuation level of the Gaussian envelope, i.e., the filter amplitude decays to $1\%$ at $\pm m_i$ or $\pm n_i$. In practice, the frequency scale and time scale are generally inversely proportional: a smaller frequency scale necessitates a larger time scale ($\sigma _{f_1} < \sigma _{f_2}$, $\sigma _{t_1} > \sigma _{t_2}$), which enables more precise frequency capture. In the special case where $\sigma _{t_1} = \sigma _{t_2}$, the center of the Gaussian filter becomes more pronounced, improving both amplitude variation detection and noise suppression, as shown in Fig. 4.

The two-dimensional Gaussian filter is applied to the previously mentioned envelope signal *en*(*n*, *t*). Assuming that the frequency domain range of the filter is $U = [u_1, u_2](m_i)$ and the time domain scales are $V = [v_1, v_2, v_3, v_4](n_i)$ ($u_1 > u_2$, $v_1> v_2> v_3 > v_4$), a total of ten filter ranges are established by combining any two time scales (which may be identical). When the time domain range is $[v_1, v_2]$ and the frequency domain range is $[u_1, u_2]$, the following two-dimensional Gaussian filter results are obtained:

9 $$\begin{aligned} D_{ij}(n,t) = \frac{ \displaystyle \sum _{s=1}^{N} \left[ \int _0^T g_{u_2v_2u_1v_1}(s, \tau ) \, en(n-s, t-\tau ) \, d\tau \right] }{ \left\| g_{u_2v_2u_1v_1}(n, t) \right\| } \end{aligned}$$

Here: *i*,*j* refers to the time scale index, derived from the set *V* (representing time domain scales); *n* is the signal channel index;$\Vert \cdot \Vert$ denotes L2 norm normalization.

The filtering results are then integrated using cross-scale contrast. Existing integration methods predominantly utilize central-surround operators to combine filtering outcomes;  however, these approaches are prone to interference^39^. Therefore, this paper proposes an optimization scheme by designing a cross-scale contrast fusion method, which allows the filtering process to adapt to multiple resolutions (in both the time and frequency domains), thereby enhancing its robustness against interference. The cross-scale contrast is defined between different filter channels as:

10 $$\begin{aligned} Q_{ijkl}(n,t) = \big (D_{ij}(n,t) - D_{kl}(n,t)\big )^2, \quad (i,j) \ne (k,l) \end{aligned}$$

where (*i*, *j*) and (*k*, *l*) represent different time-frequency filter channel combinations, derived from the set *V*. The integrated two-dimensional filtering result for the *n*-th filter channel is given by:

11 $$\begin{aligned} \varphi (n,t) = \sum _{\begin{array}{c} i,j \ne k,l \end{array}} Q_{ijkl}(n,t) \end{aligned}$$

Here: $\varphi (n,t)$ represents the integrated result of the *n*-th channel after combining all time-frequency scale combinations. To improve feature extraction in the time domain, a scale-variant one-dimensional Gaussian filter is introduced, defined as:

12 $$\begin{aligned} G(t_i) = \exp \Big (-\frac{t^2}{\sigma _{t_i}^2}\Big ) \end{aligned}$$

where $\sigma _{t_i}$ is the filtering parameter, identical to the expression in Eq. 8. The one-dimensional filtering process is similar to the two-dimensional filtering process, except for the absence of a frequency scale, retaining only the time scales from the range $V = [v_1, v_2, v_3, v_4]$. Applying $G(t_i)$ to the envelope signal *en*(*n*, *t*) yields:

13 $$\begin{aligned} D_{i}(n,t) = \frac{\int _0^T G(t_i) \, en(n,t-\tau ) \, d\tau }{\Vert G(t_i)\Vert } \end{aligned}$$

Here: *i* refers to the time scale index, derived from the set *V* (representing time domain scales); *n* is the signal channel index;$\Vert \cdot \Vert$ denotes L2 norm normalization. The cross-scale contrast for one-dimensional filtering is defined as:

14 $$\begin{aligned} Q_{im}(n,t) = \big (D_i(n,t) - D_m(n,t)\big )^2, \quad i \ne m \end{aligned}$$

Here: *i*,*m* refers to the time scale index, derived from the set *V*. The integrated one-dimensional result is given by:

15 $$\begin{aligned} \varphi '(n,t) = \sum _{\begin{array}{c} i \ne m \end{array}} Q_{im}(n,t) \end{aligned}$$

Finally, the two-dimensional and one-dimensional results are combined to take advantage of both frequency-domain and time-domain information, and the final output is:

16 $$\begin{aligned} \theta (n,t) = \varphi '(n,t) \sum _{n=1}^{N} \varphi (n,t) \end{aligned}$$

After normalization, $\theta (n,t)$ represents the final filtered signal, which provides enhanced extraction of transient components and frequency-specific features, thereby improving the overall signal quality.

### Simulation of neuronal lateral inhibition in the auditory pathway

In the case of white noise, its energy is approximately evenly distributed across frequency channels. Therefore, a simple linear suppression matrix, which operates by subtracting contributions from other channels, is generally sufficient to cancel out noise across channels. In such scenarios, a conventional suppression matrix is adequate. However, in real-world environments, noise is typically non-uniformly distributed across frequencies. Under these conditions, a conventional suppression matrix may result in insufficient suppression for channels dominated by strong noise and excessive suppression for channels with weak noise. To address this limitation, a side-improved suppression matrix *C* is introduced. This matrix performs weighted inhibition based on the inter-channel correlations, whereby channels with stronger noise exert greater suppression on other channels, while channels with weaker noise impose less suppression. This approach more accurately simulates the nonlinear dynamic compression observed in auditory pathway neurons. The detailed procedure is as follows^40^. Assuming that the frequency domain representation of the Gaussian filter *h*(*n*, *t*) is *H*(*n*, *f*), the frequency domain matrix $\Omega$ for the filter bank is defined as:

17 $$\begin{aligned} \Omega = \begin{pmatrix} H(1, f) \\ H(2, f) \\ \vdots \\ H(N, f) \end{pmatrix} \end{aligned}$$

To reflect the degree of relationship between two filters, the relationship matrix $\Psi$ is defined as:

18 $$\begin{aligned} \Psi = \Omega \Omega ^ { T } \end{aligned}$$

The compression amount for the nth channel is defined as $\theta _ { n }$:

19 $$\begin{aligned} \theta _ { n } = \sum _ { j = 1 } ^ { N } \Psi _ { n j } - \Psi _ { n n } \end{aligned}$$

where $\psi _ { n j }$ is the nth row and jth column of the $\Psi$ matrix. The side-improved suppression matrix *C *is defined as:

20 $$\begin{aligned} C = \begin{pmatrix} 0 & \Psi _{12} \theta _1^{-1} & \cdots & \Psi _{1N} \theta _1^{-1} \\ \Psi _{21} \theta _2^{-1} & 0 & \cdots & \Psi _{12} \theta _1^{-1} \\ \vdots & \vdots & \ddots & \vdots \\ \Psi _{N1} \theta _N^{-1} & \Psi _{N2} \theta _N^{-1} & \cdots & 0 \end{pmatrix} \end{aligned}$$

In the above equation, $C _ { i j }$ denotes the suppression coefficient of the jth given filter channel to the ith filter channel, from which it can be inferred that the final result $\theta _ { n } ( n, t )$ after the signal passes through n this channel is defined as:

21 $$\begin{aligned} \theta _ { n } ( n, t )= & \theta ( n, t ) - \sum _ { j = k } ^ { N } C _ { n k } \theta ( j, t ) \end{aligned}$$

22 $$\begin{aligned} \theta _ { n } ( n, t )= & \left\{ \begin{matrix} \theta _ { n } ( n, t ), i f \theta _ { n } ( n, t ) > 0 \\ 0, e l s e \end{matrix}\right. \end{aligned}$$

**Fig. 5:**
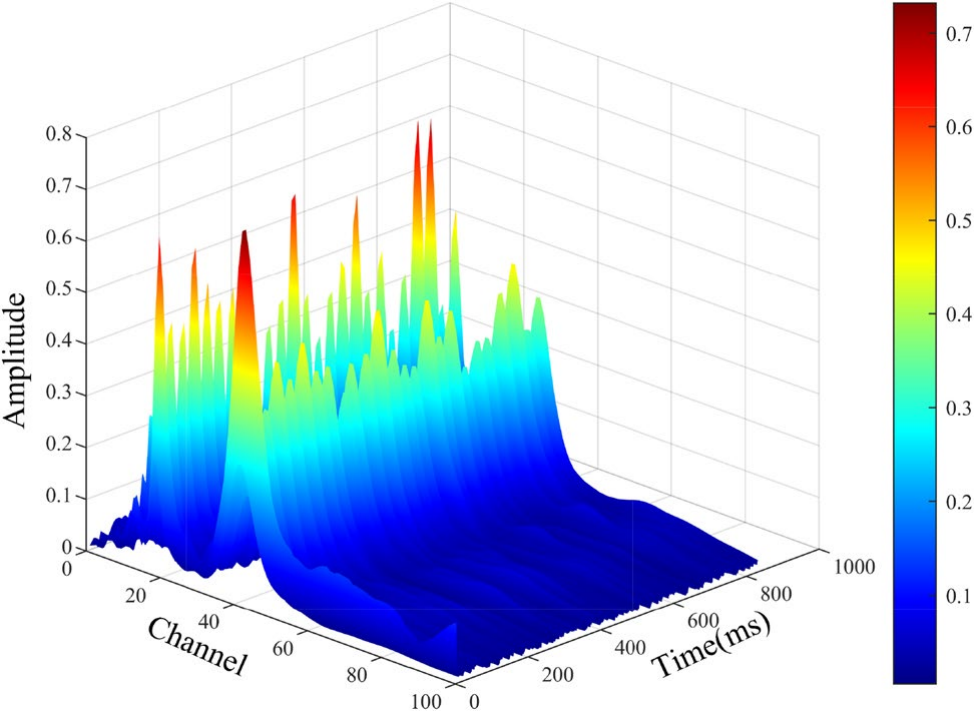
Plot of $\theta ( n, t )$ for different channels.

When a signal passes through the nth channel, the lateral suppression mechanism subtracts contributions from correlated channels (Eq. 21). This operation effectively reduces the energy of components common across channels, which usually correspond to background noise or interference. Meanwhile, unique or dominant features within the target channel, often related to actual fault or vibration signatures, are preserved. As a result, the desired signal components are relatively enhanced. The non-negativity constraint (Eq. 22) ensures that suppression does not introduce artificial negative artifacts. This constraint maintains the physical interpretability of the processed signal. The results of this process are illustrated in Fig. 5.

Through the above procedure, the processed signals for each frequency channel are obtained. These are denoted as the decomposed signals $\theta _ { n } ( n, t ) ( 1 \le n \le N )$ of the original signal *x*(*t*) at different frequencies. This operation essentially decomposes the original signal into *N* different frequency components. Next, the decomposed signal of each channel is integrated along the time axis (Perform summation for discrete values), with the resulting value representing the energy contained in the frequency signal of that channel. Finally, the results of all channels are normalized, with frequency plotted on the horizontal axis and the normalized energy values on the vertical axis. The resulting image is referred to as the spectrogram of auditory resonance peaks (RPSP). Physically, the resonant peaks represent the regions where the signal energy is concentrated, which corresponds to the peak regions in the RPSP.

### Comparative analysis with conventional methods

A signal from the Case Western Reserve University (CWRU) bearing database, with a roller fault, severity 1, and load type 1, was selected to compare RESAS and STFT^41^. Filter parameters were first determined. Prior studies show that increasing the number of filters *N* beyond 60 yields negligible improvement in performance^33^, while 100 frequency bands are sufficient for visualization and provide adequate frequency-translation intervals for 2D filtering.The duration can be set to any value without affecting the results. In the 2D filtering process, scales *V* and *U* critically influence the results: *V* defines filter time width and bandwidth, and *U* determines the number of filtering channels. Parameter selection considers (1) the high frequency overlap of Gabor transform (GT) filters, allowing fine frequency resolution, and (2) the wide frequency range of transient signals. Accordingly, four *V* scales cover 10, 20, 30, and 40 channels, while two *U* scales cover 4 and 6 channels.

**Fig. 6:**
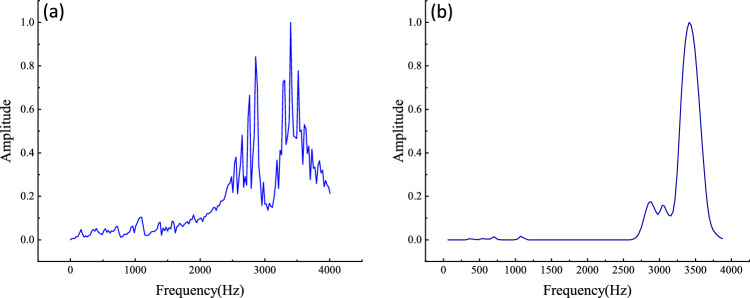
Comparison chart of the two methods (**a**) Traditional STFT (1D resonance peak via time-axis summation); (**b**) RPSP of RESAS.

The comparison presented in Fig. 6 demonstrates that the RESAS method provides a more refined spectral envelope of the signal. In contrast to the traditional short-time Fourier transform (STFT), which sums the magnitude along the time axis to obtain a 1D resonance peak plot, the RESAS method not only preserves and enhances the information contained in high-frequency resonance peaks (main resonance peaks), but also effectively suppresses low-frequency peaks and spurious peaks. Additionally, the RESAS method eliminates the burrs caused by abrupt changes at the time-window boundaries, thereby improving peak contrast and enhancing the overall quality of the resonance spectrum. Compared to the traditional approach, the RESAS method exhibits superior performance^42^.

## Two-layer random forest model (TL-RF)

**Fig. 7:**
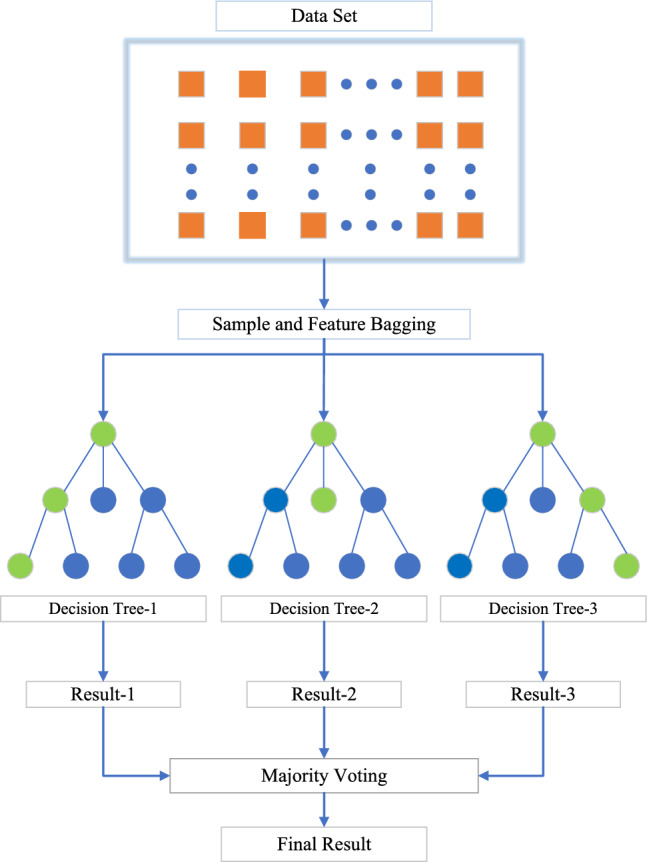
Random forest model diagram.

Random Forest is an ensemble learning method primarily used for classification and regression tasks. The fundamental idea behind the Random Forest model is to introduce diversity among decision trees by utilizing different subsets of data and features for each tree. This ensures that the ensemble does not overly rely on any specific tree, thereby improving its ability to generalize to unseen data. Specifically, the Random Forest architecture leverages non-parametric resampling to generate decorrelated training sets for each tree, where instance-level randomness injects diversity while mitigating the risk of overfitting. At each node, the Greedy Algorithm is employed to determine the optimal split. The data is sorted in ascending order, and all possible binary splits are evaluated. The split with the lowest Gini coefficient is selected as the optimal split point. Among all features, the one yielding the smallest Gini coefficient after evaluating the optimal split for all features is chosen as the splitting feature at that node^43^. The overall framework of the Random Forest model is illustrated in Fig. 7, which provides a depiction of the complete process.

23 $$\begin{aligned} G i n i ( t ) = 1 - \sum _ { k = 1 } ^ { k } P _ { k } ^ { 2 } \end{aligned}$$

where $\text {Gini}(t)$ denotes the Gini coefficient at node *t*, $P_k$ is the proportion of class *k* at the node, and *k* is the total number of classes. Repeating this process *n* times produces a Random Forest model consisting of *n* decision trees. The final prediction is obtained by majority voting across all trees, which can be formally expressed as:

24 $$\begin{aligned} P _ { j } = \frac{ 1 }{ n } \sum _ { j = 1 } ^ { n } y _ { j } ^ { i } \end{aligned}$$

$P _ { j }$ is the probability of classifying the input into the j-th class. $y _ { j } ^ { i }$ is the prediction outcome, where in the i-th decision tree classifies the input into the j-th class.

**Fig. 8:**
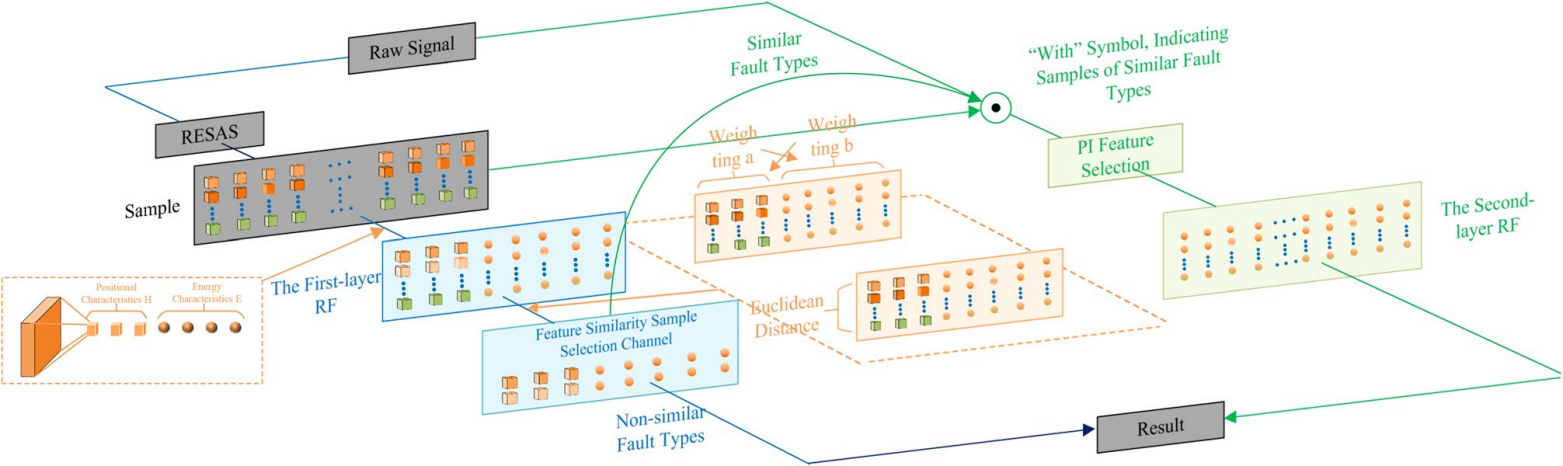
TL-RF.

Traditional single-layer RF model exhibit several drawbacks. When handling complex nonlinear feature interactions, it may be difficult to model them effectively and distinguish useful features. Secondly, It is relatively sensitive to noise and outliers in small samples, making them susceptible to data quality issues. Thirdly, It may be biased toward majority classes, diminishing predictive performance for minority classes. Lastly, The computation speed will be slow when the number of samples or features is too large. To address the aforementioned limitations, this paper proposes a two-layer random forest model (TL-RF), as shown in Fig. 8. The model comprises two layers of RF networks interconnected by an intermediate feature similarity-based sample selection channel. Hierarchical modeling significantly enhances feature representation, thereby improving the model’s ability to capture complex nonlinear interactions between features.

Firstly, the vibration signal *x*(*t*) is processed through RESAS to obtain the RPSP representation. Peak values corresponding to the first three highest amplitudes are extracted, and their associated channel indices are denoted as $H _ {1}, H _ {2}, H _ {3}$, The position feature set is thus defined as *H*(*x*). In addition, the RPSP is partitioned into five regions along the channel axis (100 channels in total, 20 per region). The summed amplitudes in each region yield the energy features $E _ {1}, E _ {2},..., E _ {5}$, which is denoted as the energy feature *E*(*x*) Subsequently, the first-layer random forest is trained using both position and energy features. The number of trees ($f _ { 1 }$) is determined from the out-of-bag (OOB) error curve, while the Gini index is employed as the splitting criterion to ensure purer nodes^44^. A minimum subset size of five samples is adopted to terminate splitting, and a 70/30 train–test split is applied to balance efficiency and accuracy.

To further refine discrimination between fault types, a feature similarity–based sample selection mechanism is introduced. First, since the position features *H* and energy features *E* differ significantly in scale, weighting is applied: coefficients *a* and *b* are set to the mean values of *H* and *E*, respectively. After weighting, the feature set of a signal sample *P* becomes $\left[ H _ { 1 p }, H _ { 2 p }, H _ { 3 p }, E _ { 1 p }, E _ { 2 p }, E _ { 3 p }, E _ { 4 p }, E _ { 5 p } \right]$ After weighting, the feature set becomes $\left[ a H _ { 1 p }, a H _ { 2 p }, a H _ { 3 p }, b E _ { 1 p }, b E _ { 2 p }, b E _ { 3 p }, b E _ { 4 p }, b E _ { 5 p } \right]$ . The values of the weighting coefficients *a* and *b* are defined as:

25 $$\begin{aligned} a= & \frac{ \sum _ { k = 1 } ^ { 5 } E _ { k p } }{ 5 } \end{aligned}$$

26 $$\begin{aligned} b= & \frac{ \sum _ { k = 1 } ^ { 3 } H _ { k p } }{ 3 } \end{aligned}$$

$H _ { k p }$ the k-th bit of the positional signature of signal; $E _ { k p }$ is the k-th bit of the energy signature of signal *p*. The introduction of this weighting mechanism ensures that different features are processed on the same scale, preventing model instability caused by scale differences. This process also serves to reduce noise and enhance model robustness.

Secondly, The features of each fault type signal are represented by calculating the average features of the sample signals within each fault type. Assuming that the number of samples for each fault signal is M and considering a fault type A, the features of this fault type signal are represented as $\left[ H _ { 1 } ^ { A }, H _ { 2 } ^ { A }, H _ { 3 } ^ { A }, E _ { 1 } ^ { A }, E _ { 2 } ^ { A }, E _ { 3 } ^ { A }, E _ { 4 } ^ { A }, E _ { 5 } ^ { A } \right]$, where $H _ { 1 } ^ { A }$ is defined as Eq. 27, and the other features are obtained similarly. By calculating the characteristics of each fault type and evaluating the Euclidean distance between different faults, we can theoretically quantify the similarity between fault types. Smaller distances indicate higher similarity.

27 $$\begin{aligned} H _ { 1 } ^ { A } = \frac{ \sum _ { P = 1 } ^ { M } H _ { 1 p } }{ M } \end{aligned}$$

Assuming there are two fault types, A and B, their feature sets are represented as$\left[ H _ { 1 } ^ { A }, H _ { 2 } ^ { A }, H _ { 3 } ^ { A }, E _ { 1 } ^ { A }, E _ { 2 } ^ { A }, E _ { 3 } ^ { A }, E _ { 4 } ^ { A }, E _ { 5 } ^ { A } \right]$ and $\left[ H _ { 1 } ^ { B }, H _ { 2 } ^ { B }, H _ { 3 } ^ { B }, E _ { 1 } ^ { B }, E _ { 2 } ^ { B }, E _ { 3 } ^ { B }, E _ { 4 } ^ { B }, E _ { 5 } ^ { B } \right]$ respectively. The Euclidean distance between them is denoted as $\varepsilon ( A, B )$.

28 $$\begin{aligned} \varepsilon ( A , B ) = \sqrt{ \sum _ { k = 1 } ^ { 3 } ( H _ { k } ^ { A } - H _ { k } ^ { B } ) ^ { 2 } + \sum _ { k = 1 } ^ { 5 } ( E _ { k } ^ { A } - E _ { k } ^ { B } ) ^ { 2 } } \end{aligned}$$

Finally, the threshold for $\varepsilon ( A, B )$ is established, whereby features with distances below this threshold are defined as similar features. Subsequently, samples containing these similar features are imported into the second-layer random forest. This feature similarity-based sample selection mechanism enables us to distinguish between different types of fault signals with greater accuracy, thereby enhancing the diagnostic precision of the model.

**Fig. 9:**
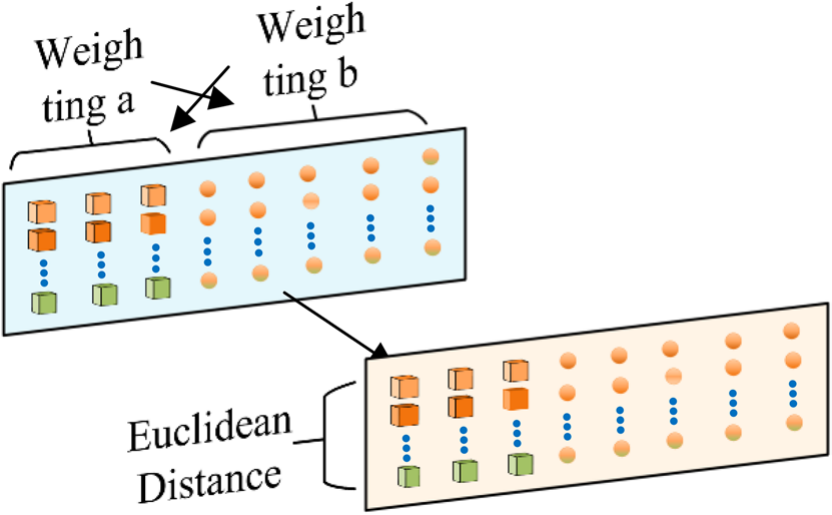
Feature similarity selection channel.

In the second-layer network, the amplitude values of all channels are used as training features to preserve detailed information. The number of trees is set to the larger value between the OOB-optimal tree number and 1.5 times the feature count, while other settings remain consistent with the first layer. Feature importance is subsequently quantified using Permutation Importance (PI), which measures prediction accuracy changes when individual features are randomly permuted. This method is more intuitive than Gini-based importance, as it directly reflects the actual contribution of features to model performance^45^. Finally, a threshold is applied to remove uninformative or detrimental features, and the refined feature set is used to construct the second-layer random forest. The overall process is illustrated in Fig. 9.

### Diagnostic process

**Fig. 10:**
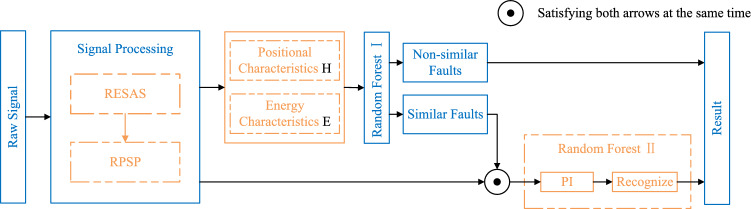
Fault discrimination flowchart.

The signal to be recognized is initially processed through the RESAS model to generate the RPSP, from which relevant features are extracted and input into the first layer of the random forest. If the output indicates feature similarities associated with faults, the RPSP is passed to the second-layer random forest via a feature similarity sample selection channel, where its fault category is determined through the second-layer network. Conversely, if the first-layer random forest produces distinct discriminative results, those results are directly output. Finally, a threshold is applied to assess feature importance, and the remaining features are used to construct the second-layer random forest. The detailed process is illustrated in Fig. 10.

### QPZZ-II comprehensive fault simulation test bench data verification

**Fig. 11:**
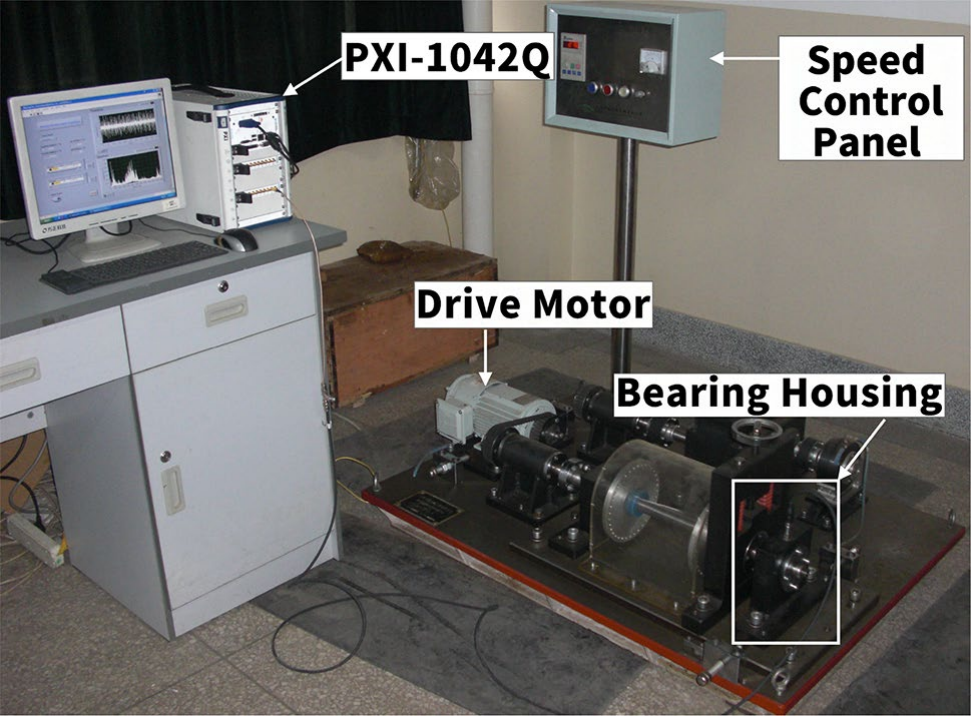
QPZZ-II comprehensive fault simulation test bench.

The QPZZ-II comprehensive fault simulation test bench (Fig. 11) is designed for rotating machinery and can simulate various faults, including bearing faults, gear faults, rotor imbalance, and misalignment. As shown in the image, the platform mainly consists of a driving motor, a shaft system supported by two bearing housings, a loading motor, and a speed control panel. One of the bearing housings is detachable, allowing convenient replacement of bearings with different fault types. In addition, a transparent protective cover is mounted around the shaft to ensure safety while allowing direct observation of the rotor and bearings during operation.

**Fig. 12:**
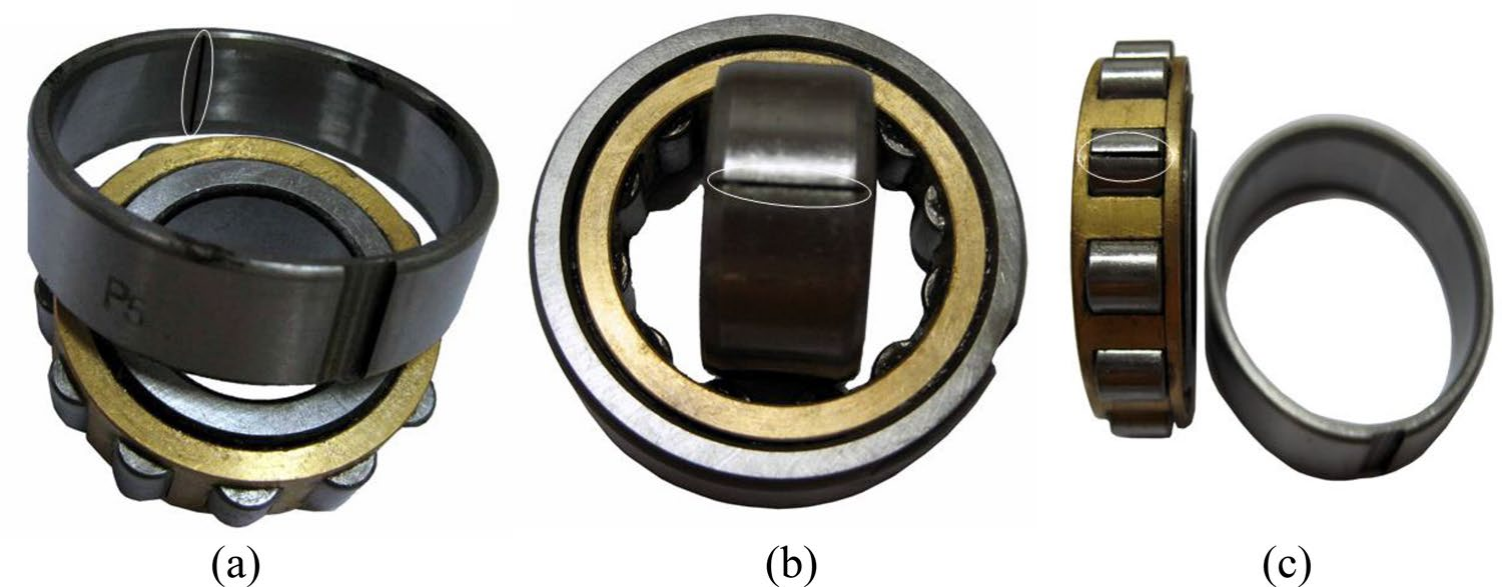
Bearing failure pictures: (**a**) Outer ring failure (**b**) Inner ring failure (**c**) Roller failure.

Vibration signals were collected using a PCB M603C01 ICP acceleration sensor, which integrates a charge amplifier for compactness and easy installation. Data acquisition and storage were performed with the NI PXI-1042Q high-performance testing system, which incorporates a PXI-4472 data acquisition card and LabVIEW software. The system supports up to 16 parallel acquisition channels, with a maximum sampling rate of 102.4 kHz. In this study, the sampling frequency was set to 12,000 Hz, and 120,000 data points were collected for each fault type. The experiment focused on four bearing states: normal, outer race fault, inner race fault, and rolling element fault (Fig. 12). NU205 cylindrical roller bearings were employed, with localized defects introduced into the outer ring, inner ring, and rolling elements via wire-cutting technology. The defect dimensions were $15 \times 0.5 \times 0.5~\text {mm}$. The rotor was supported at both ends by NU205 bearings, with the faulty bearing mounted in the detachable right bearing housing. Tests were conducted under two operating speeds: 800 $\mathrm{{r}}/\mathrm{{min}}$ and 1396 $\mathrm{{r}}/\mathrm{{min}}$.Specific hardware and software parameters, along with specific bearing parameters, are detailed in Tables 1 and 2.

**Table 1:** Experimental setup for bearing test bench.

Test bench	Sensor	Rolling bearing	Data acquisition system
QPZZ-II comprehensive fault simulation test bench for rotating machinery	PCB M603C01 ICP accelerometer (USA)	NU205 cylindrical roller bearing	NI PXI-1042Q high-performance acoustic and vibration testing system

**Table 2:** Geometric parameters of the rolling bearing.

Pitch diameter (mm)	Rolling element diameter (mm)	Number of rolling elements	Contact angle ($^{\circ }$)
39	7.5	12	0

For model input preparation, vibration signals at 800 $\mathrm{{r}}/\mathrm{{min}}$ were divided into 12 equal segments per fault type, resulting in 12 samples per condition, which were then used for training the proposed RESAS model.

**Fig. 13:**
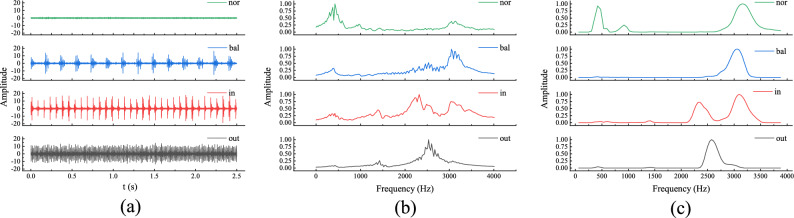
Laboratory signal map (**a**) Raw signal; (**b**) STFT; (**c**) RPSP.

Figure 13a shows the raw signals of the bearing under four states at 800 rpm. Figure 13b presents the envelope of the signal after its short-time Fourier transform (STFT). Figure 13c illustrates the RPSP, further demonstrating the ability of the RESAS to highlight and envelope the resonance peaks. The parameters of the RESAS model are selected in Table 3 below:

**Table 3:** RESAS model parameters.

Parameter	Value	Parameter	Value	Parameter	Value
*N*	100	$f_h$	12000	*v*	0.0959
$u_1$	1.86396	$u_2$	2.79594	$v_1$	4.65991
$v_2$	9.31981	$v_3$	13.97972	$v_4$	18.63962

**Fig. 14:**
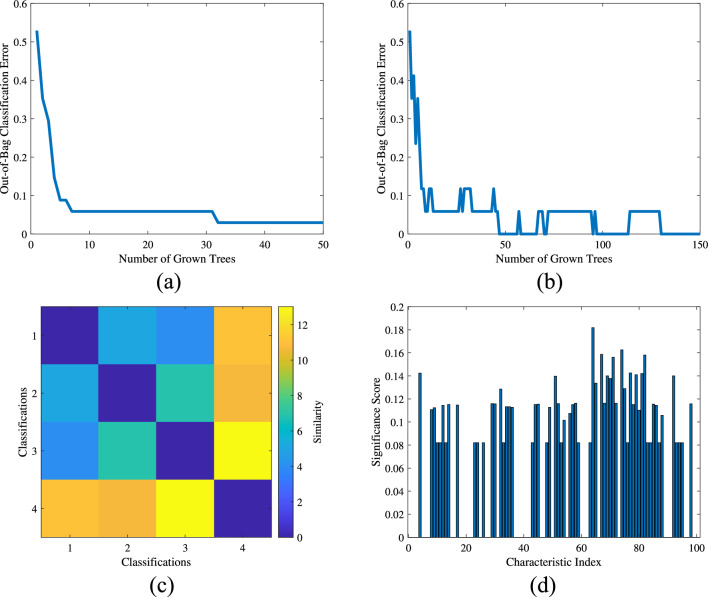
TL-RF model parameter diagram (**a**) Plot of first layer network accuracy vs. number of trees; (**b**) Plot of second layer network accuracy vs. number of trees; (**c**) Plot of feature similarity; (**d**) Plot of PI feature importance analysis.

Next, the obtained results were fed into the TL-RF for training. Figure 14a illustrates the relationship between the accuracy of the first-layer random forest and the number of trees. The horizontal axis represents the number of random forest trees, while the vertical axis indicates the corresponding accuracy. From the figure, it can be observed that selecting 50 trees is relatively reasonable. The training method for the second-layer random forest is similar to that of the first layer, as shown in Fig. 14b. However, due to the number of features involved, the number of trees in the second layer is set to 150. Figure 14c presents the similarity analysis between features in the first-layer random forest. The similarity threshold is set to 3, and the similar faulty features identified are 1 and 3. Figure 14d shows the importance analysis results of features in the second-layer random forest after PI training. The importance threshold is set to 0.1, and the detailed configuration of all network parameters is provided in the Table 4.

**Table 4:** TL-RF parameter list.

Number of first-layer $f_1$	Similarity threshold	Number of second-layer $f_2$	PI threshold
50	150	3	0.1

**Fig. 15:**
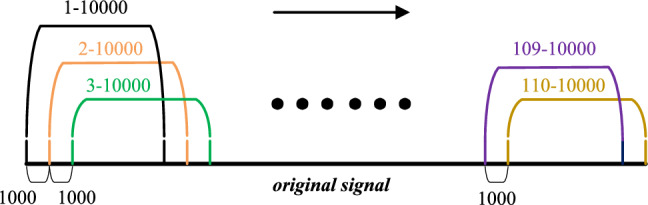
Fault discrimination flowchart.

To validate the fault identification performance, each fault signal category was processed in segments at its own rotational speed of 800 $\mathrm{{r}}/\mathrm{{min}}$. The length of each fault signal category was set to 10,000. For each segmentation, the frame shift was set to 1000, and the overlap length was set to 9000, Fig. 15 illustrates the segmentation scheme. 110 data sets were extracted from each signal segment, resulting in a total of 440 data sets for detection.

**Fig. 16:**
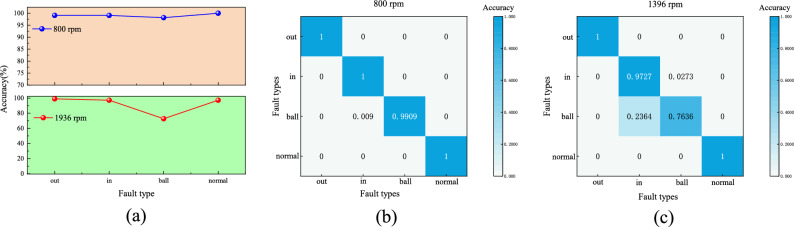
(**a**) Accuracy of bearing inspection at different rotational speeds (**b**) Confusion Matrix at 800 rpm (**c**) Confusion Matrix at 1396 rpm.

As shown in Fig. 16, subfigure (a) presents the classification accuracy under two operating conditions. The upper panel corresponds to the 800 rpm fault identification experiment, where the accuracy exceeds 99%, confirming the effectiveness of the proposed model in bearing signal evaluation. Subfigure (b) displays the corresponding confusion matrix for 800 rpm. It can be seen that the model accurately distinguishes between all fault categories, including inner-ring, outer-ring, and ball defects, with minimal misclassification. This indicates that the feature extraction and classification performance is highly reliable under this condition. Subsequently, a transfer experiment was conducted using samples with a rotational speed of 1396 rpm, processed in the same manner as the 800 rpm samples. The 1396 rpm samples were pre-processed using the same signal denoising, feature extraction, and normalization techniques, yielding 440 test sets. The classification accuracy at 1396 rpm, shown in the lower panel of Fig. 16a, remains above 90%, which demonstrates the model’s robustness when subjected to varying rotational speeds. However, the confusion matrix in Fig. 16c reveals a few misclassifications, particularly between the outer-ring and ball faults. This can be attributed to the similarity in their vibration signatures under different load conditions, as well as the changes in spectral features induced by the variation in rotational speed. Despite this, the detection accuracy for both outer- and inner-ring faults still exceeds 95%, highlighting the model’s strong transferability and stability across different operating conditions.

## Data detection using the case Western Reserve University dataset

### Verify fault recognition

**Fig. 17:**
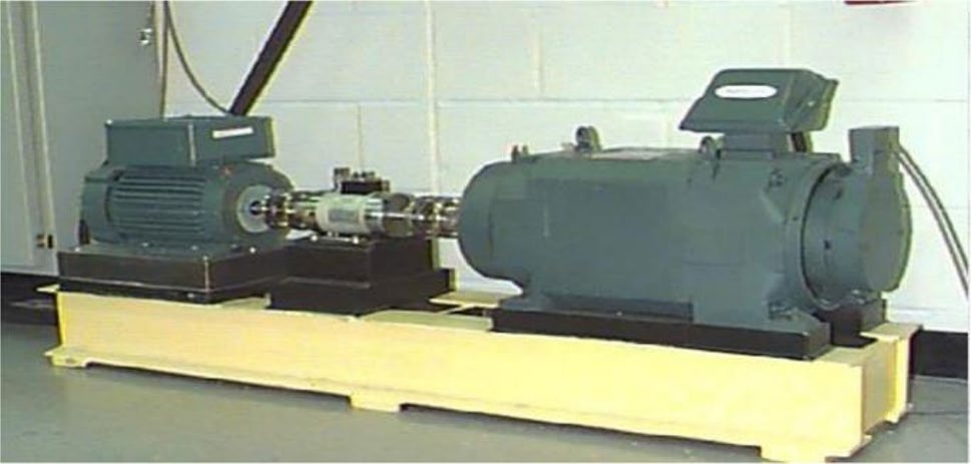
Case Western Reserve University lab bench.

The experiment utilized the experimental data provided by Case Western Reserve University for the validation of vibration signals in the model. The experiment was conducted on a simulated test bench, An accelerometer was positioned at the Drive End to collect vibration signals, with a sampling frequency of 12 *kHz*^46^. The experimental setup illustrated in Fig. 17 represents a data acquisition and measurement system, featuring a 2-horsepower induction motor (left position), a torque transducer integrated with an optical encoder (central assembly), and a hydraulic dynamometer (right terminus), arranged in a linear configuration. Control electronics are schematically omitted from the diagram.

During the experimental process, defects were artificially introduced into the outer race, inner race, and rollers by the researchers. Various degrees of fault were represented by different defect depths. To simulate the operational states of bearings under different working environments, the motor was operated under varying loads (0–3 hp) and speeds (1730–1797 rpm). A load condition of 1 hp was selected for this study. The damage depths were set at 0.1778 mm, 0.3556 mm, and 0.5334 mm, corresponding to damage levels denoted by the numbers 1, 2, and 3, respectively. The damage locations on the outer race were positioned at the 3 o’clock, 6 o’clock, and 12 o’clock directions, represented by CE, OR, and OP, respectively. In the subsequent text and tables, numerical labels ranging from 1 to 14 are utilized for representation. The Table 5 presents a summary of the data selected for this study:

**Table 5:** Summary table of bearing failure types.

	Out CE			Out OR		Out OP	
Severity	1	2	3	1	3	1	3
Tab	131	198	235	145	247	158	259
Nicknames	1	2	3	4	5	6	7

	In			Ball			Normal
Severity	1	2	3	1	2	3	–
Tab	106	170	210	119	186	223	98
Nicknames	8	9	10	11	12	13	14

**Fig. 18:**
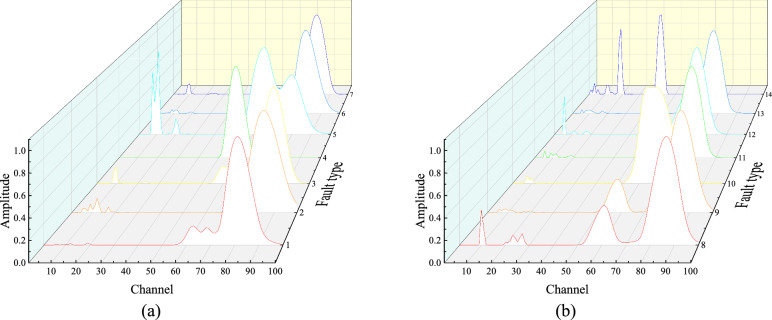
RPSP for different fault types under load of 1 of the Case Western Reserve University Dataset (**a**) Failure 1–7; (**b**) Failure 8–14.

**Fig. 19:**
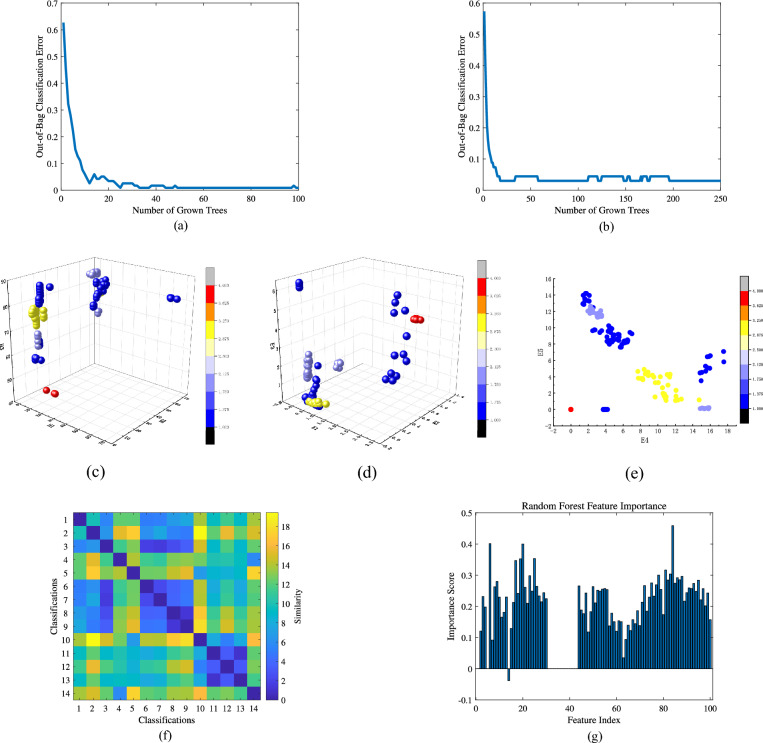
TL-RF model parameter diagram (**a**) Plot of the first layer network accuracy versus the number of trees; (**b**) Plot of the second layer network accuracy versus the number of trees; (**c**) Distribution of feature $H_{1}, H_{2}, H_{3}$; (**d**) Distribution of feature $E_{1}, E_{2}, E_{3}$; (**e**) Distribution of feature $H_{4}, H_{5}$; (**f**) Distribution of feature similarity relationship; (**g**) PI feature importance analysis.

Similar to the data processing method described for the Test Bench above, each type of fault signal was first divided into 12 equal segments, resulting in 12 samples per fault type and a total of 168 sample groups. These samples were then input into the RESAS model, with the model parameters selected as specified in Table 3. Figure 18 shows the RPSP for faults 1 to 14, respectively. Subsequently, the results were fed into the TL-RF for training. The parameter selection method was consistent with the aforementioned approach. The relationship between the accuracy of the first-layer random forest and the number of trees is illustrated in Fig. 19a, indicating that selecting 50 trees is appropriate. Figure 19b shows that the number of trees of the second-layer random forest is set to 150. Figure 19c–e present the distribution of fault features of the first-layer random forest. Different fault types are represented by distinct numbers and colors: outer race faults are denoted by 1, inner race faults by 2, rolling element faults by 3, and normal conditions by 4. It can be observed from the figures that features extracted from signals with the same fault type share strong statistical similarities, and variations in fault depth further lead to sub-clusters within the same fault category. Figure 19f shows the similarity between detailed fault features, with a threshold set at 3. Fault features with similar characteristics are numbered 3, 6, 7, 8, 9, 11, 12, and 13. Figure 19g displays the importance of different features after PI training in the second-layer random forest, with the threshold set at 0.1. All parameters are detailed in Table 6.

**Table 6:** TL-RF parameter List.

First-layer $f_1$	Similarity threshold	Second-layer $f_2$	PI threshold
100	250	3	0.1

**Fig. 20:**
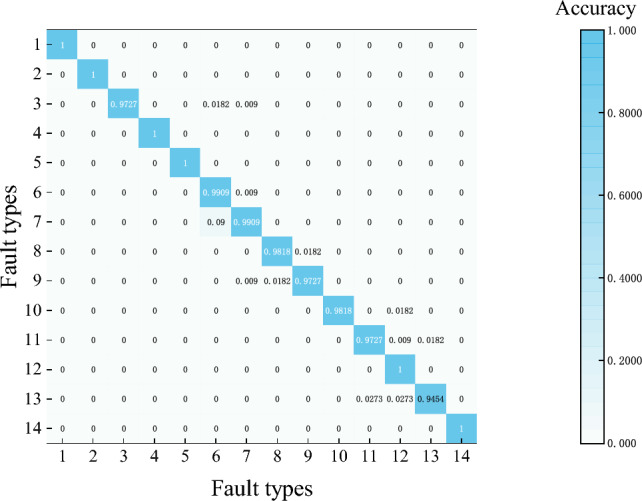
Confusion matrix.

To evaluate model performance, various fault signals were segmented into 10,000-frame intervals. Each segmentation employed a 1000-frame shift with 9000-frame overlap strategy, yielding 110 data sets per segment. Ultimately, 1540 data sets were used as the test set to assess the model’s fault classification capability. The model classification results are shown in Fig. 20. Diagnostic evaluation via the confusion matrix indicates that the model demonstrates significant efficacy in bearing defect detection. Its machine learning framework achieves an overall classification accuracy of 98.64% across all operating conditions.

### Stability and anti-interference capability

**Fig. 21:**
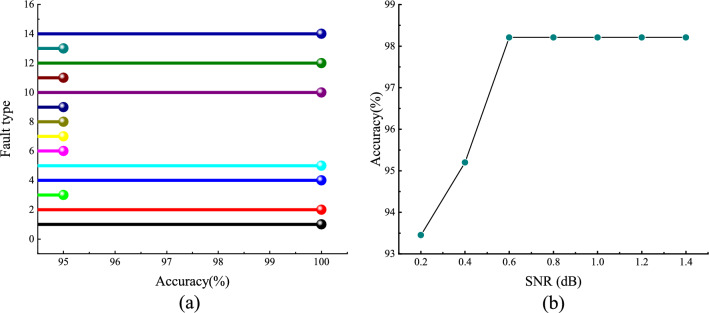
(**a**) Accuracy of different length tests; (**b**) Accuracy at different signal-to-noise ratios.

To evaluate the model’s stability and the influence of signal length on validation performance, 20 additional groups of signals were extracted from the dataset. Signal lengths varied from 5000 to 15,000 samples in increments of 500, with a frame shift of 3000 samples applied for each group. The corresponding accuracy rates are shown in Fig. 21a. As illustrated, the overall accuracy remains around 98%, with approximately half of the samples achieving 100% accuracy. These results indicate that signal length has minimal effect on detection performance, demonstrating the model’s high stability. Furthermore, the model’s robustness against noise was evaluated, given the complex and often unknown noise characteristics in bearing vibration signals. Gaussian white noise of varying variances was added to the fault signals, which were segmented into 20 portions each, yielding a total of 280 signals. The unprocessed original signals were used as templates for testing, and the accuracy of fault diagnosis was subsequently calculated to assess noise interference resilience.

This approach effectively assesses the model’s noise resistance capability. The accuracy after testing is shown in Fig. 21b. The data indicate that as the signal-to-noise ratio (SNR) increases, the accuracy improves to a certain extent, reaching a peak after 0.6 dB. However, the overall accuracy consistently remains above 93%, demonstrating that the model possesses robust anti-noise capabilities.

#### Applications under different operating conditions

**Fig. 22:**
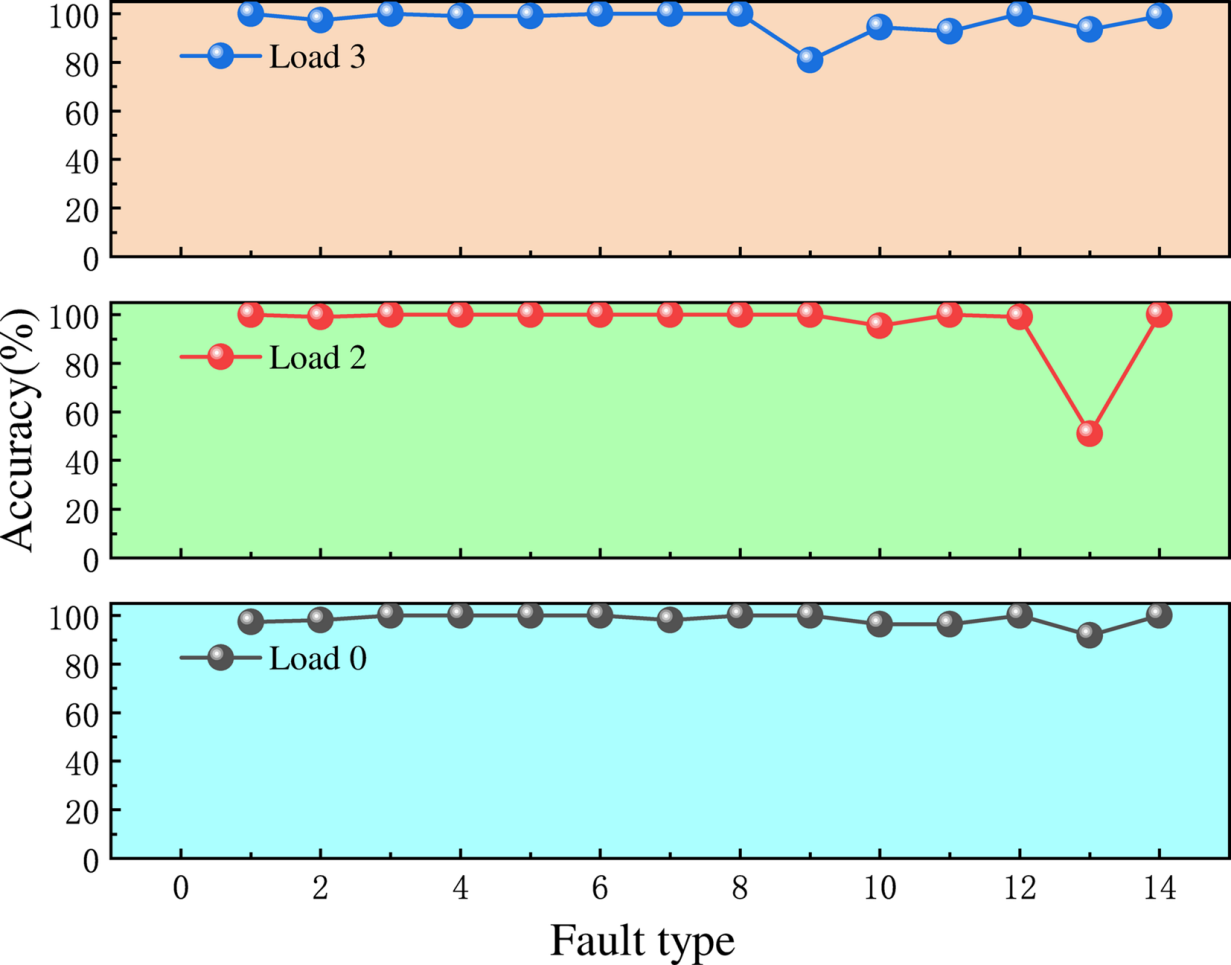
Accuracy of migration test under different loads.

To further assess the sensitivity of the model to different operating conditions and to determine its capability for transfer validation. We applied identical processing to the test signals under various load conditions, including 0 hp, 2 hp, and 3 hp. For each load condition, 110 segments of data with a length of 10,000 were extracted, resulting in a total of 4,620 data groups for evaluation. The evaluation results are presented in Fig. 22.

As shown in Fig. 22, under different load conditions, the fault identification accuracy for most fault types exceeds 95%. Specifically, the verification accuracy for fault types 1, 4, 5, 6, and 8 reaches 100% across all load conditions, and the overall accuracy is 96.7%. However, fault type 13 under load condition 2 exhibits a relatively lower verification accuracy. Detailed analysis indicates that the features of fault type 13 are highly similar to those of fault type 11, which belongs to the same major fault category, thereby affecting the classification results. Notably, the detected fault was classified as type 11, which still falls within the rolling element fault category. In summary, for transfer experiment detection, the proposed method maintains high accuracy, and the fault templates can be applied across different operating conditions, demonstrating considerable practical applicability.

### Comparison of the proposed model

**Fig. 23:**
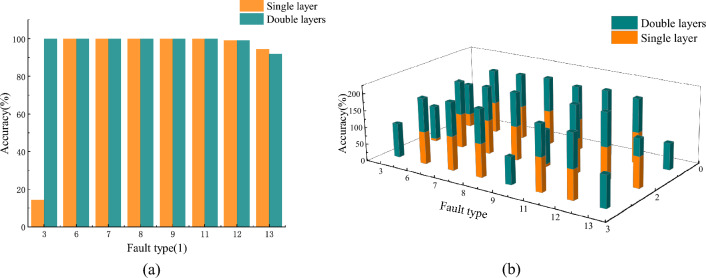
(**a**) Accuracy comparison of the same group with different model architectures; (**b**) Accuracy of 0, 2, 3 migration tests with different model architecture loading.

To validate the necessity and superiority of the TL-RF proposed in this study, a layer random forest test was conducted on the aforementioned signals, and the two models were compared. The first-layer random forest only forwards samples with fault types 3, 6, 7, 8, 9, 11, 12, and 13 (similar feature faults) to the second-layer random forest. Consequently, only the accuracy rates of these specific fault types are affected. Comparisons were made exclusively for these cases, and the resulting accuracy rates are presented in Fig. 23a and b.

It can be observed that a single-layer random forest allows clear identification of most fault types under identical operating conditions, achieving high accuracy. However, the accuracy for fault type 3 is relatively low. This occurs because the first-layer random forest selects features based on the positions of several prominent peaks and the sum of multi-channel amplitudes, while neglecting other peak values and the amplitude features of individual channels. As a result, the features of fault types 3 and 7 become highly similar, causing the single-layer random forest to struggle in distinguishing between them. To address this issue, a second-layer random forest was introduced to further differentiate these fault types by utilizing the previously overlooked feature details.

**Fig. 24:**
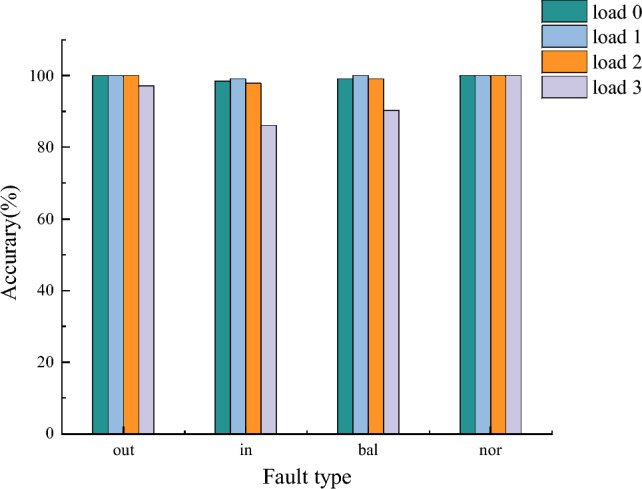
Accuracy of major fault type classification under different model architectures with multiple loads.

In transfer validation under different operating conditions, fault type 3 was largely misclassified under loads of 2 and 3 hp, fault type 9 exhibited lower recognition accuracy under the same loads, and fault type 13 was completely misclassified under loads of 0 and 3 hp. Overall, the accuracy of the single-layer random forest is considerably lower compared to the TL-RF model. Single-layer random forests only consider fault type detection accuracy, as shown in Fig. 24.

When conducting fault detection under identical operating conditions or focusing solely on specific fault types, the accuracy of a single-layer random forest is sufficient. However, to enhance the generalizability of the method and improve the accuracy of transfer validation, this study employs the TL-RF dual-layer random forest network model.

### Comparison with other methods

**Table 7:** Identification accuracy of different fault diagnosis methods under the same group test.

Detection methods	Out	In	Ball	Normal	Number of identifications	Availability of migration
BIC	98.8%	95.2%	98.3%	100%	4	Cannot
NCA^47^	99.6%	97.2%	99.6%	98.0%	4	Cannot
PCA^47^	99.6%	93.6%	96.8%	97.2%	4	Cannot
MobileNet-V2^48^	98.3%	99.3%	97.0%	100%	10	Cannot
RESAS+ TL-RF	99.4%	97.9%	97.3%	100%	16	Can

**Table 8:** Recognition accuracy of different fault diagnosis methods under transfer testing.

Methods	1–0	1–2	1–3
TCA^49^	92.50%	93.06%	87.05%
JDA^49^	92.78%	95.28%	88.61%
CNN^49^	92.50%	93.06%	88.22%
DANN^50^	94.42%	92.56%	96.60%
RESAS+ TL-RF	97.31%	96.12%	96.88%

To demonstrate the superiority of the RESAS+ TL-RF model, the fault diagnosis accuracy for bearings was compared with other methods. The comparison results for the vibration signal dataset are presented in Tables 7 and 8.

1. A more detailed classification of fault types is provided by RESAS+ TL-RF compared to Blind deconvolution Laplace wavelet analysis(BIC), Neighborhood component analysis(NCA), and Principal component analysis(PCA), with higher accuracy being achieved. Compared to MobileNet-V2, six additional fault types are considered, maintaining comparable accuracy. Furthermore, transferability testing can be performed, offering greater practical value.
2. When compared with models under other variable operating conditions, a significant improvement in accuracy is observed with RESAS+TL-RF. Its average accuracy is increased by 5.9% over the TCA model, by more than 4.55% over the JDA model, by 5.51% over the CNN model, and by 3.44% over the DANN model.
3. Compared to CNN-based methods, the parameter selection process is easier in RESAS+TL-RF, and it is more convenient for practical applications.

## Discussion of the results

The RESAS+ TL-RF model demonstrated strong performance in fault detection and classification for bearing systems. In experiments with the Case Western Reserve University dataset, the model achieved over 99% accuracy, highlighting its effectiveness under controlled conditions. When tested at a different rotational speed (1396 rpm), the model maintained an impressive overall recognition rate above 90%, with specific accuracy for outer- and inner-ring faults exceeding 95%, demonstrating its robust transferability. Comparison with other methods, including BIC, NCA, PCA, and MobileNet-V2, showed that the RESAS+ TL-RF model not only outperformed these techniques in accuracy but also offered the advantage of transfer learning, which enhanced its practical value. Additionally, it outperformed other transfer learning methods (TCA, JDA, DANN) in fault classification accuracy, confirming its superior robustness in variable operating conditions. The model also proved stable across varying signal lengths and resilient to noise, maintaining high accuracy (over 93%) even in noisy conditions. These results underline the model’s reliability, robustness, and generalizability, making it highly applicable for real-world bearing fault diagnosis.

## Conclusion

Inspired by the auditory attention mechanism of the human ear, this study introduces a novel resonance peak extraction method, termed RESAS.This method employs three key components: Gamma Tone (GT) filtering, multi-scale Gaussian filtering, and lateral inhibition matrix processing, which together approximate the functionality of the human auditory system. These components efficiently extract the resonance peaks and construct a resonance peak salience map (RPSP). Subsequently, the features extracted from the RPSP are used as inputs to a TL-RF model for fault classification. The main research findings and contributions are summarized as follows:

- The proposed method was validated using both experimental data from QPZZ-II Comprehensive Fault Simulation Test and publicly available data from the Case Western Reserve University Bearing Data Center. Results demonstrate that the method can reliably identify bearing defect location and severity. Moreover, it exhibits stable performance under varying rotational speeds and load conditions, indicating strong generalization ability and reduced need for repeated retraining.
- Impulsive mechanical faults often induce variations in system eigenfrequencies. Due to the broadband characteristics of such signals, resonance peaks are naturally excited. The proposed method enhances the visibility of these peaks, suggesting potential applicability to a broader range of shock-related fault diagnosis tasks.
- By simulating the auditory processing pathway through biologically inspired filtering and inhibition mechanisms, the method constructs a resonance peak saliency map that improves resonance peak localization and separation. This framework provides a new perspective for signal representation in mechanical fault diagnosis.
- Although high-precision instruments were used for controlled validation in this study, the method does not fundamentally rely on costly hardware. With recent advances in low-cost MEMS sensors and embedded signal processing, RESAS can be feasibly applied in practical civilian scenarios such as electric vehicles, thus extending its potential for real-world deployment.

## Supplementary Information

Supplementary Information.

## Data Availability

The datasets used in this study are publicly available from the Case Western Reserve University Bearing Data Center. The data can be accessed at: https://engineering.case.edu/bearingdatacenter/download-data-file.
