# Supplementary material for: Resonance peak extraction method based on human ear model and its application in bearing fault diagnosis
Source: Sci Rep. 2025 Nov 25;15:41932. doi: 10.1038/s41598-025-25794-8 (PMC12647139; doi:10.1038/s41598-025-25794-8)
Supplement: Supplementary file 1 — Supplementary Information. [file 41598_2025_25794_MOESM1_ESM.pdf]

In order to verify the effectiveness of the method in this paper, a four-degree-of-freedom bearing simulation model is established in the following, and different faults are modeled and simulated, and the results are analyzed.

Bearing Composition

Rolling bearings typically consist of four components: the inner ring, the outer ring, rolling elements, and the cage. Several dynamic models have been developed to date, including Fukata's dynamic model, Sopanen and Mikkola's model, and the MATLAB-Simulink bearing model[1–3], each of which is suitable for different scenarios. For example, the Fukata model addresses the nonlinear and time-varying characteristics of rolling bearings, but it is a two-degree-of-freedom model. The model proposed by Sopanen and Mikkola considers both distributed defects (such as waviness in the inner and outer rings) and local defects (such as faults in the inner and outer rings), with MSC.ADAMS being employed for the implementation and analysis of the ball bearing model. Although the results are promising, the model is considered overly complex and computationally demanding. The model employed below is inspired by the MATLAB-Simulink bearing model, with certain aspects being simplified to meet simulation requirements, thereby enabling the simulation of the actual bearing operation.

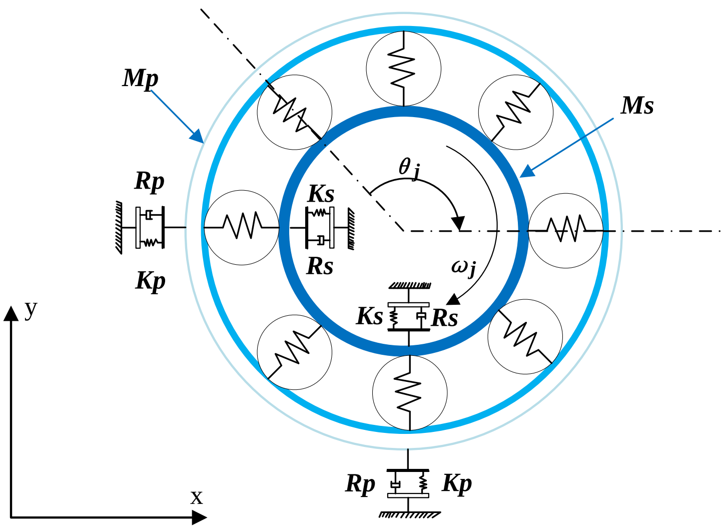

Figure 1. Bearing kinematics model

The model accounts for the inertia of the inner and outer rings, the contact forces, and the impact of localized faults. During the development of the model, the inertial effects of the cage and rolling elements are assumed to be negligible. It is assumed that the rolling elements are arranged at equal angular intervals in a specific manner and rotate together with the cage at a constant angular velocity. To simulate external conditions and enhance the vibration effects, a constant force Wx is applied in the x-direction of the bearing's inner ring. The model has four degrees of freedom, with two degrees of freedom for both the inner and outer rings. The rotor rotates at a constant angular velocityω_c, ω_s representing the output shaft angular velocity. M_P, K_P and R_P represent the supporting mass, spring stiffness, and damping of the outer ring, respectively. Similarly, M_S, K_S and R_S represent the mass, stiffness, and damping of the inner ring, respectively. The outer ring is connected to the base via a spring-damping system. The model is developed based on the following assumptions:

(1) The stationary outer race remains kinematically constrained, whereas the inner race maintains a uniform angular velocity under steady-state operation.

(2) Surface interactions (between the balls and the inner and outer rings) are assumed to follow the Hertz contact model.

(3) Continuous contact is maintained between the balls and the base, and no sliding occurs.

(4) The balls and the cage are assumed to be massless, and all translational motion is confined to the x-y plane, while rotational motion occurs only about the z-axis.

Table 1. Bearing model parameters

Parameter	Value
Mass of pedestal (M_p)	12.638
Stiffness of pedestal (K_p)	15.1056×10^6
Damping of pedestal (R_p)	2210.7
Mass of shaft (M_s)	6.638
Stiffness of shaft (K_s)	4.241×10^4
Damping of shaft (R_s)	1376
Ball diameter (d)	7.94
Pitch diameter (D)	39.32
Fault width (w)	0.4
Number of balls (n)	9
Radial clearance (c)	5×10^{-3}

Table 2. Kinematic Characteristics of Bearing Components

Parameter	Value
Rotor angular velocity (ω_c)	$41.711\,\mathrm{rad\,s^{-1}}$
Output shaft angular velocity (ω_s)	$104.720\,\mathrm{rad\,s^{-1}}$
Ball pass frequency outer race (BPFO)	$3.591 f_s$
Ball pass frequency inner race (BPFI)	$5.409 f_s$
Ball spin frequency (BSF)	$1.577 f_s$
Fundamental train frequency (FTF)	$0.399 f_s$
Initial angular position (θ_j)	0 rad

Rolling bearing parameters

Hz represents the average number of contact occurrences between the rolling elements and the inner or outer rings. The relationships between the various motion parameters can be defined as[3]:

$$\theta_j = \frac{2\pi(j-1)}{N_b} + \omega_c t + \theta_0 \tag{1}$$

$$\omega_c = \frac{\omega_s}{2}\left(1 - \frac{d}{D}\right) \tag{2}$$

$$BPFI = \frac{n_b f_s}{2}(1 + \frac{d}{D}) \tag{3}$$

$$BPFO = \frac{n_b f_s}{2}(1 - \frac{d}{D}) \tag{4}$$

$$BSF = \frac{f_s}{2}(\frac{D}{d})(1 - (\frac{d}{D}))^2 \tag{5}$$

Equations of Motion

The deformation and position of the j-th rolling element are denoted asδ_j, θ_j respectively. The formulas for the Hertzian contact forces in the horizontal direction (F_x) and vertical direction (F_y) are defined as[4]:

$$F_x = \sum_{j=1}^{N_b} K_b \delta_j^{\gamma} \cos\theta_j \cdot h(-\delta_j) \tag{6}$$

$$F_y = \sum_{j=1}^{N_b} K_b \delta_j^{\gamma} \sin\theta_j \cdot h(-\delta_j) \tag{7}$$

first, the equivalent contact stiffness K_b. is calculated, which is define as:

$$k_b = \left[\frac{1}{(1/k_{bi})^{\frac{2}{3}} + (1/k_{bo})^{\frac{2}{3}}} \right]^{\frac{3}{2}} \tag{8}$$

here, k_{bi} and k_{bi} represent the contact stiffness between the rolling elements and the inner ring, respectively. They are defined as:

$$k_{bi} = \frac{2\sqrt{2}}{3} (\frac{E}{1-\mu^2})(\delta_i^*)^{-\frac{3}{2}} (\sum \rho)_i^{-\frac{1}{2}} \tag{9}$$

$$k_{b0} = \frac{2\sqrt{2}}{3} (\frac{E}{1-\mu^2})(\delta_o^*)^{-\frac{3}{2}} (\sum \rho)_o^{-\frac{1}{2}} | \tag{10}$$

$\delta_i^* \cdot$ and $\delta_o^* \cdot$ are denoted as the contact displacement coefficients of the rolling elements, the inner and outer rings, respectively. E is represented as the elastic modulus of the material. $\mu \cdot$ signifies the Poisson's ratio of the material, which reflects the lateral contraction ratio in other directions when the material is subjected to tensile stress.$(\sum \rho)_i$ and $(\sum \rho)_0$ are defined as the sums of curvatures at the contact points of the inner and outer rolling elements, respectively. These curvature sums are determined by the geometric properties of the rolling elements and the inner and outer raceways. They are defined as:

$$(\sum \rho)_i = \rho_{b1} + \rho_{b2} + \rho_{i1} + \rho_{i2} \tag{11}$$

$$(\sum \rho)_o = \rho_{b1} + \rho_{b2} + \rho_{o1} + \rho_{o2} \tag{12}$$

where P represents curvature; b, o, i represent roller, outer ring, inner ring respectively; 1,2 represent radial, axial respectively. for example, $\rho_{b1} \cdot$ represents the curvature of roller radial direction.

$$\rho_{b1} = \rho_{b2} = \frac{2}{D_b} \tag{13}$$

$$\rho_{i1} = \frac{2}{D-d} \tag{14}$$

$$\rho_{i2} = -\frac{1}{\delta_i^* d} \tag{15}$$

$$\rho_{o1} = -\frac{2}{D+d} \tag{16}$$

$$\rho_{o2} = -\frac{1}{\delta_0^* d} \tag{17}$$

Subsequently, the $\gamma \cdot$ value in the equation is determined by the type of bearing (here taken as 3/2). The value of $\cdot h(x)$ is given as[5]:

$$h(x) = \begin{cases} 1, & x \geq 0 \\ 0, & x < 0 \end{cases} \tag{18}$$

the deformation $\delta_j \cdot$[5] is defined as:

$$\delta_j = x_d \cos\theta_j + y_d \sin\theta_j - c \tag{19}$$

in the formula, $\cdot x_d$, $\cdot y_d$ denotes the horizontal and vertical displacements between the inner and outer rims and the ball, respectively, when the failure occurs. The deformation $\delta_j \cdot$ disappears and becomes zero when it is less than 0. The motion of the inner ring of the bearing can be defined as:

$$M_P \ddot{x}_0 = F_x - R_P \dot{x}_0 - K_P x_0 \tag{20}$$

$$M_p \ddot{y}_0 = F_y - M_p g - R_p \dot{y}_0 - K_p y_0 \tag{21}$$

$$M_s \ddot{x}_i = Wx - F_x + R_s \dot{x}_i + K_s x_i \tag{22}$$

$$M_s \ddot{y}_i = -F_y - M_s g - R_s \dot{y}_i - K_s y_i \tag{23}$$

where x, y represent horizontal and vertical respectively; 0,i represent outer and inner circle centers of mass respectively; $\dot{x}$, $\ddot{x}$ represent velocity and acceleration respectively; for example, $\ddot{x}_i$ denotes the acceleration in the horizontal direction of the outer circle center of mass, g is the acceleration taken as $10\ \mathrm{m/s^2}$, and the rest of the variables are given in table 1.

Fault Modeling

The fault types are divided into three categories: outer ring, inner ring, and the ball. The total displacement δ_o[6] resulting from the fault follows the same formula, but the calculation of the displacement δ_f differs in the specific details. The following sections will introduce each fault type separately.

$$\delta_o = \max(x_d \cos\theta_j + y_d \sin\theta_j - c - \delta_f, 0) \tag{24}$$

$$x_d = x_i - x_0 \tag{25}$$

$$y_d = y_i - y_0 \tag{26}$$

In the first case, when a fault occurs in the outer ring, the rolling element continuously collides with the damaged area of the outer ring (here, a notch is used to represent the actual form of damage), generating cyclical impacts during the bearing's rotation. As shown in the figure, when the roller enters the pit, its displacement changes, causing an impact, and when it exits, the displacement returns to zero. Therefore, its displacement δ_f is expressed as Eq: 29, (where C_d is the local displacement, and C_{dr} represents the fault depth).

$$C_{dr} = \frac{1}{2(d - \sqrt{d^2 - 4w^2})} \tag{27}$$

$$C_d = C_{dr} \tag{28}$$

$$\delta_f = \begin{cases} C_d, & \text{if } \phi_d < \theta_j < \phi_d + \Delta\phi_d \\ 0, & \text{else} \end{cases} \tag{29}$$

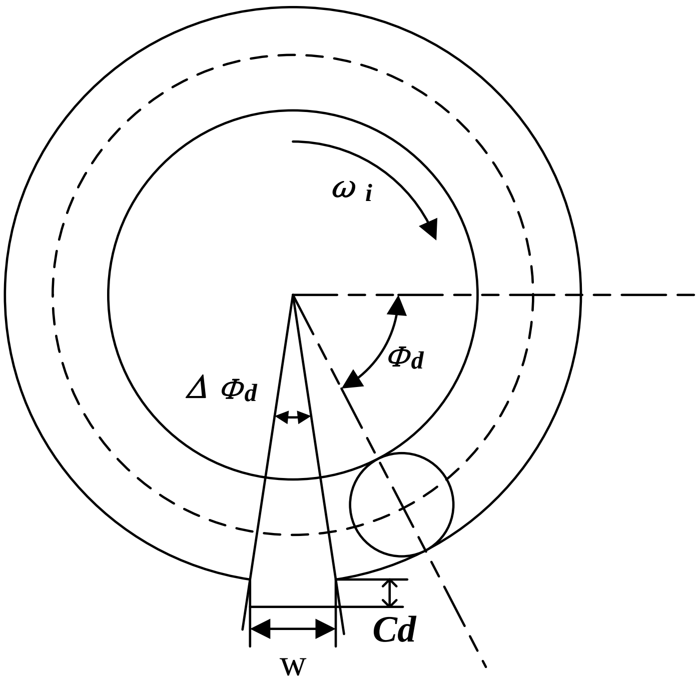

Figure 2. Bearing outer ring failure diagram

In the second scenario, when a fault occurs in the inner ring, similar to the outer ring case, the ball continuously collides with the inner ring, generating impacts. However, unlike the first case, this impact is related to the rotational speed of the shaft. The displacement δ_f is determined in the same way as Eq 29.

$$\phi_d = \omega_c t + \phi_0 \tag{30}$$

In the third scenario, when a roller experiences damage, as shown in the schematic diagram, the recessed area rotates along with the roller. Its position can be described by the following formula. Each full rotation results in one collision with both the inner and outer rings, generating impacts. The resulting angular width for each collision is defined as:

$$\phi_s = \frac{\omega_c D}{2d}(1-(\frac{d}{D}\cos\phi)^2)t + \phi_0 \tag{31}$$

$$\phi_{bi} = \frac{\Delta\phi_d d}{D_i} \tag{32}$$

$$\phi_{bo} = \frac{\Delta\phi_d d}{D_0} \tag{33}$$

here, D_i and D_0 represent the inner and outer rings of the bearing, respectively, and are related to D, d, c in Table 2 as follows:

$$D_i = \frac{D-d}{2} - c \tag{34}$$

$$D_0 = \frac{D+d}{2} + c \tag{35}$$

during the rolling process, when the roller contacts the inner ring, the roller moves downward while the inner ring moves upward. The total local displacement, denoted as C_d is the sum of both displacements. The displacement of the inner ring is

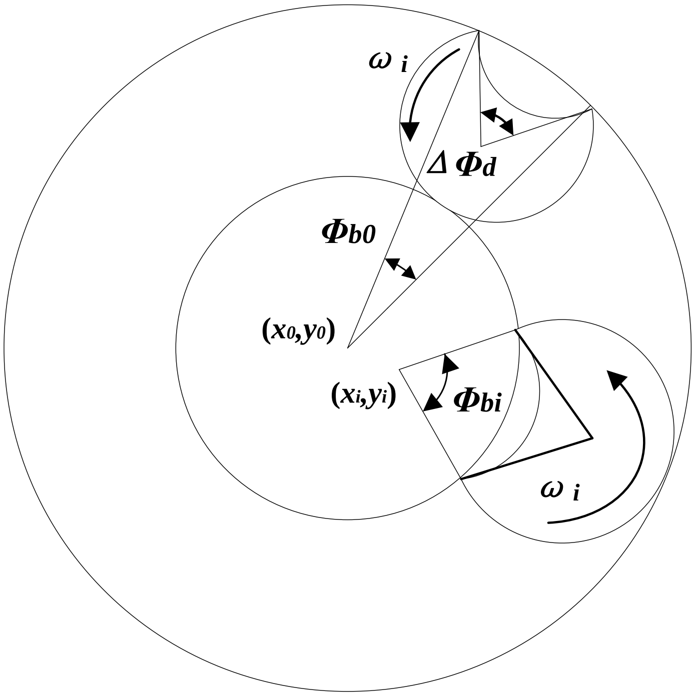

Figure 3. Bearing Roller Failure Diagram

denoted as C_{di}, which is defined as:

$$C_{di} = \frac{1}{2(D_i - \sqrt{D_i^2 - 4w^2})} \tag{36}$$

C_d is defined as:

$$C_d = C_{dr} + C_{di} \tag{37}$$

similarly, during the contact between the roller and the outer ring, a local displacement also occurs. However, since they both move downward during contact, $C_d$represents the difference between the two displacements. The displacement of the outer ring is denoted as C_{d0}, and is defined as:

$$C_{do} = \frac{1}{2(D_0 - \sqrt{D_0^2 - 4w^2})} \tag{38}$$

C_d is defined as:

$$C_d = C_{dr} - C_{do} \tag{39}$$

the total displacement δ_f is defined as:

$$\delta_f = C_d = \begin{cases} C_{dr} - C_{do}, & \text{if } 0 < \phi_s < \phi_{bo} \\ C_{dr} + C_{di}, & \text{if } \pi < \phi_s < \pi + \phi_{bi} \\ 0, & \text{else} \end{cases} \tag{40}$$

Simulation and Analysis

The fault function is incorporated into the dynamic equations to solve for the system's behavior. To achieve a faster response, certain initial parameters are assigned to the bearing. The outer and inner rings are given initial displacements of 1×10^{-6} in both the x-directions. The resulting speed response of the inner ring under different fault conditions is then calculated.

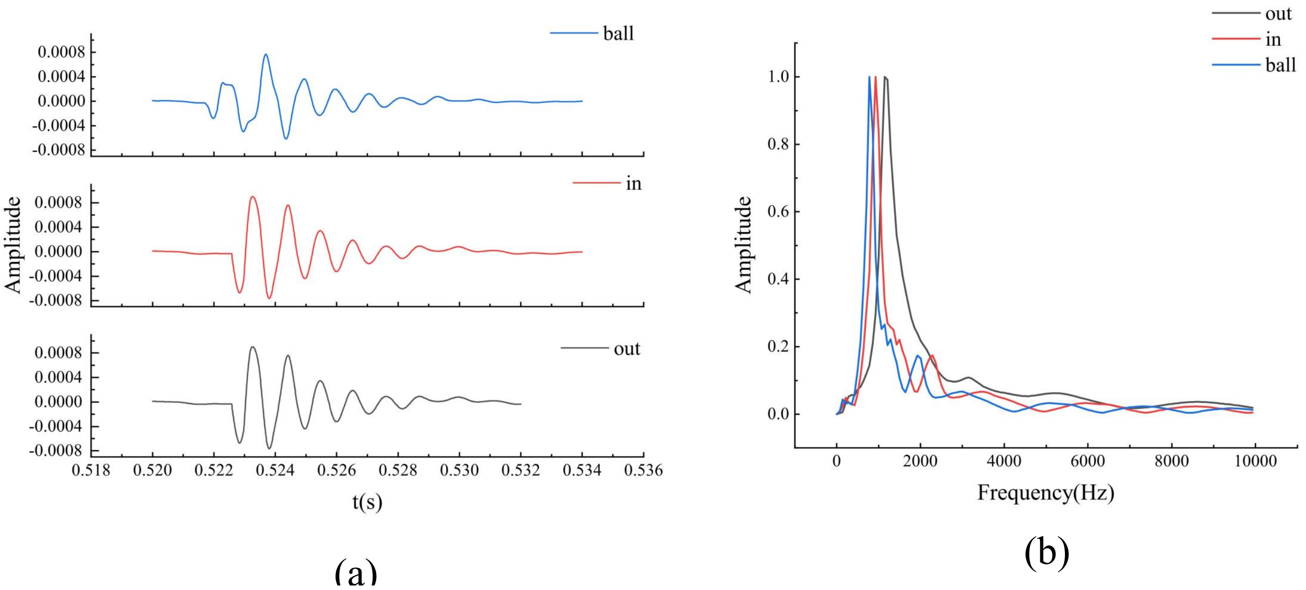

Figure 4. Time-frequency diagram of a single shock signal (a) Time domain; (b)Frequency domain

Figure. 4(a) illustrates the single-impact waveforms for three fault types. The waveforms of the inner and outer rings are similar, characterized by a sharp rise to the peak followed by a gradual decay to a steady state. In contrast, the rolling element waveform shows a slow increase and gradual decrease. This difference arises because the fault point on the outer ring is stationary, leading to "instantaneous" impact loading as the rolling element passes, which produces a "spike" waveform. Although the fault point on the inner ring rotates with the shaft, its rapid rotation results in an "instantaneous" impact signal, similar to the "spike" of the outer ring. In contrast, the rolling element fault point experiences a smoother loading and unloading process, producing a "hump" shape. Figure 4(b) displays the corresponding normalized frequency spectrum. The outer ring shows one peak, while the inner ring and the rolling body each show two peaks, with significant differences between the three peaks.

In practical detection, fault shock signals may be non-periodic or irregular, and it is difficult to extract the signal directly in the time domain, making it impractical to use a single shock signal for detection. Next, multiple shocks are analyzed, and conventional processing typically uses the Short Time Fourier Transform (STFT) to average the spectrum and suppress random noise. Essentially, this involves statistically analyzing the eigenfrequencies of the shock signal between the time and frequency domains, i.e., the resonance peaks. Fig. 5 displays the waveform diagrams of three fault types under multiple impacts. from which it can be seen that the outer ring impacts do not change with time, and each impact is the same, which is because the outer ring is fixed and the condition of each impact is the same. The measured interval between consecutive impacts is 0.0167 s(59.880 Hz) that closely matches the characteristic frequency of 59.746 Hz. Upon waveform amplification, a double-impact phenomenon can be observed. The formula of Δt is Eq. 41. The theoretical impact interval is calculated as 0.00172 s, while the experimentally determined interval measures 0.00182 s, yielding an error magnitude of 5.8%. This discrepancy may be attributed to measurement system tolerances and environmental vibration interference.

$$\Delta t = \frac{2\arcsin(\frac{\omega}{D_0})}{w_c(1-\frac{d}{D})} \tag{41}$$

For the inner ring faults, the interval between consecutive impacts is measured as 0.0114s (87.919 Hz), which closely aligns with the characteristic frequency of 90.254 Hz. Distinct from outer ring impacts, periodic variations in impact characteristics are observed. This phenomenon is attributed to the cyclic positional relationship between the defect zone and the applied load under constant operating conditions. The impact intensity varies systematically, manifesting as periodic amplitude modulation in the waveform, with the modulation period governed by the rotational frequency of the shaft. In rolling element faults, impact events are separated by 0.0252s (39.683 Hz), demonstrating strong consistency with the theoretical characteristic frequency of 39.279 Hz. Although the waveform morphology resembles inner ring fault patterns, more pronounced amplitude modulation is evident. These agreements validate the simulation accuracy.

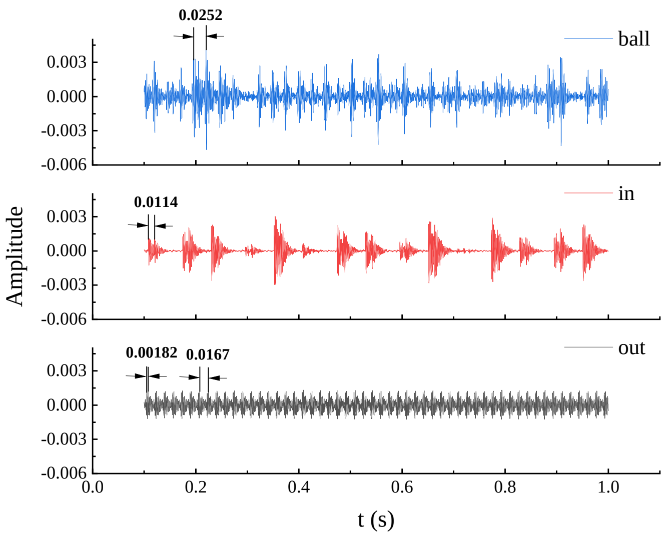

Figure 5. Time-domain waveform of the signal under multiple shocks

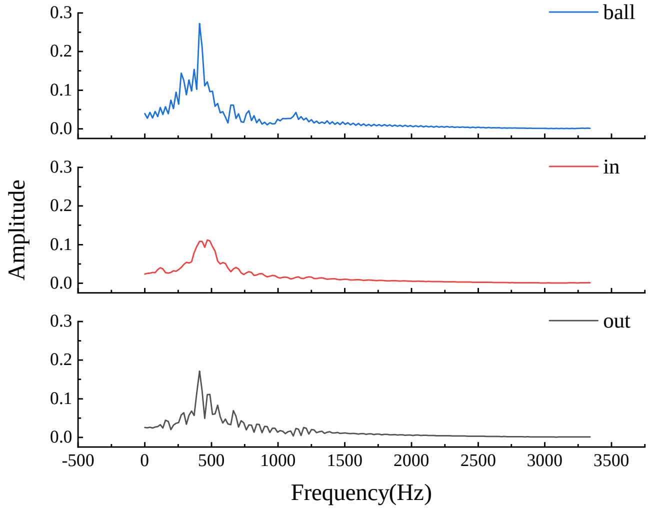

Figure 6. Spectrogram of the signal under multiple shocks

In order to facilitate better observation of the resonance peaks, we have enveloped the STFT results and obtained the spectral envelope as shown in Fig6. From the figure, it can be observed that the patterns of the three faults are similar to that of a single impact, and there are large differences between the three, so they can be utilized to differentiate the faults. However, the overall image shows many spurious peaks. This is due to the mismatch between the window length chosen for the FFT transform and the period of the impact, resulting in spectral leakage. Therefore, the GTSPF resonance peak extraction method is proposed in the main text to improve it.

References

1. S. Fukata, E. H. Gad, T. Kondou, T. Ayabe, H. Tamura, On the radial vibration of ball bearings: computer simulation, *Bull. JSME* **28**(239) (1985) 899–904, https://doi.org/10.1299/jsme1958.28.899.
2. J. Sopanen, A. Mikkola, Dynamic model of a deep-groove ball bearing including localized and distributed defects, *J. Sound Vib.* **217** (2003) 213–223, https://doi.org/10.1243/14644190360713560.

3. C. Mishra, A. K. Samantaray, G. Chakraborty, Ball bearing defect models: a study of simulated and experimental fault signatures, *J. Sound Vib.* **400** (2017) 86–112, https://doi.org/10.1016/j.jsv.2017.04.010.

4. T. A. Harris, M. N. Kotzalas, *Rolling Bearing Analysis: Essential Concepts of Bearing Technology*, 5th ed., CRC Press, New York, 2006, https://doi.org/10.1201/9781420006599.

5. N. Sawalhi, R. B. Randall, Simulating gear and bearing interactions in the presence of faults: Part I. The combined gear bearing dynamic model and the simulation of localised bearing faults, *Mech. Syst. Signal Process.* **22**(8) (2008) 1924–1951, https://doi.org/10.1016/j.ymssp.2007.12.001.

6. Y. C. Yu, T. C. Wang, T. C. Shih, A comprehensive finite-element human ear model to estimate noise-induced hearing loss associated with occupational noise exposure, *Comput. Methods Programs Biomed.* **226** (2022) 107179, https://doi.org/10.1016/j.cmpb.2022.107179.
